# The sucrose–trehalose 6-phosphate (Tre6P) nexus: specificity and mechanisms of sucrose signalling by Tre6P

**DOI:** 10.1093/jxb/ert457

**Published:** 2014-01-13

**Authors:** Umesh Prasad Yadav, Alexander Ivakov, Regina Feil, Guang You Duan, Dirk Walther, Patrick Giavalisco, Maria Piques, Petronia Carillo, Hans-Michael Hubberten, Mark Stitt, John Edward Lunn

**Affiliations:** ^1^Max Planck Institute of Molecular Plant Physiology, Am Mühlenberg 1, 14476 Potsdam-Golm, Germany; ^2^Dipartimento di Scienze e Tecnologie Ambientali Biologiche e Farmaceutiche, Seconda Università degli Studi di Napoli, Via Vivaldi, 43 I-81100 Caserta, Italy

**Keywords:** Arabidopsis thaliana, signalling, sucrose, translation, trehalose-6-phosphate, trehalose-phosphate phosphatase, trehalose-phosphate synthase.

## Abstract

Trehalose 6-phosphate (Tre6P), the intermediate of trehalose biosynthesis, has a profound influence on plant metabolism, growth, and development. It has been proposed that Tre6P acts as a signal of sugar availability and is possibly specific for sucrose status. Short-term sugar-feeding experiments were carried out with carbon-starved *Arabidopsis thaliana* seedlings grown in axenic shaking liquid cultures. Tre6P increased when seedlings were exogenously supplied with sucrose, or with hexoses that can be metabolized to sucrose, such as glucose and fructose. Conditional correlation analysis and inhibitor experiments indicated that the hexose-induced increase in Tre6P was an indirect response dependent on conversion of the hexose sugars to sucrose. Tre6P content was affected by changes in nitrogen status, but this response was also attributable to parallel changes in sucrose. The sucrose-induced rise in Tre6P was unaffected by cordycepin but almost completely blocked by cycloheximide, indicating that *de novo* protein synthesis is necessary for the response. There was a strong correlation between Tre6P and sucrose even in lines that constitutively express heterologous trehalose-phosphate synthase or trehalose-phosphate phosphatase, although the Tre6P:sucrose ratio was shifted higher or lower, respectively. It is proposed that the Tre6P:sucrose ratio is a critical parameter for the plant and forms part of a homeostatic mechanism to maintain sucrose levels within a range that is appropriate for the cell type and developmental stage of the plant.

## Summary

Trehalose-6-phosphate is a signal of sucrose status in plants and forms part of a homeostatic mechanism that maintains sucrose levels within a range that is appropriate for the cell type and stage of development.

## Introduction

Trehalose 6-phosphate (Tre6P) is the intermediate of trehalose biosynthesis and an essential signal metabolite in plants and fungi (Paul *et al.*, 2008). Tre6P is synthesized from UDP-glucose (UDPG) and glucose 6-phosphate (Glc6P) by trehalose-phosphate synthase (TPS) and dephosphorylated by trehalose-phosphate phosphatase (TPP) to yield trehalose, which can be hydrolysed to glucose by trehalase (Cabib and Leloir, 1958). Loss of TPS activity in the *Arabidopsis thaliana tps1* null mutant leads to embryo arrest at the torpedo stage (Eastmond *et al.*, 2002; Gómez *et al.*, 2006). The *tps1* mutant can be rescued by dexamethasone-inducible or embryo-specific expression of TPS1, but the resulting plants are stunted and late flowering (van Dijken *et al.*, 2004; Gómez *et al.*, 2010; Wahl *et al.*, 2013). In maize, loss of the RAMOSA3 isoform of TPP leads to abnormal inflorescence branching (Satoh-Nagasawa *et al.*, 2006). Heterologous expression of bacterial or fungal TPS and/or TPP also affects leaf morphology, photosynthetic activity, leaf senescence, and abiotic stress tolerance (Romero *et al.*, 1997; Pilon-Smits *et al.*, 1998; Garg *et al.*, 2002; Wingler *et al.*, 2012).

Constitutive overexpression of the *Escherichia coli* TPS and TPP in *A. thaliana* indicated that Tre6P, rather than trehalose, is responsible for many of the growth and developmental defects (Schluepmann *et al.*, 2003). Overexpression of TPS gave rise to early-flowering plants with small leaves and highly branched inflorescences, whereas overexpression of TPP resulted in late-flowering plants with large leaves and less branched inflorescences. The late-flowering phenotype of the embryo-rescued *tps1* null mutant is due to near loss of *FLOWERING LOCUS T* expression in the leaves, while perturbation of trehalose metabolism in shoot apical meristem cells leads to precocious flowering, acting via the miR156/SPL pathway (Wahl *et al.*, 2013). Tre6P inhibits sucrose-non-fermenting-1-related protein kinase (SnRK1) in developing tissues (Zhang *et al.*, 2009; Debast *et al.*, 2011; Nunes *et al.*, 2013*a*), potentially affecting growth and other cellular processes (Baena-González *et al.*, 2007).

To understand the physiological functions of Tre6P, we need to know what determines its level in plant cells. Lunn *et al.* (2006) developed a highly sensitive assay for Tre6P using anion-exchange high-performance liquid chromatography coupled to tandem mass spectrometry (LC-MS/MS). This assay is highly specific because it incorporates three sequential filters: baseline separation in the LC phase of Tre6P from its most common isomer, sucrose 6’-phosphate, and selection of specific parent and fragmentation product ions in the first and third quadrupoles of the mass spectrometer. Reliability was assured by: (i) spectrophotometric calibration of Tre6P standards; (ii) spiking with a deuterated-Tre6P internal standard to allow correction for ion suppression; and (iii) demonstration of acceptable recoveries (>80%) during tissue extraction. LC-MS/MS-based assays have become the method of choice for measuring Tre6P in plant tissues (Veyres *et al.*, 2008; Debast *et al.*, 2011; Delatte *et al.*, 2011; Sastre Toraño *et al.*, 2012).

Carbon (C)-starved *A. thaliana* seedlings contain very low Tre6P (0.018 nmol g^–1^ of fresh weight (FW)), but within 15–30min of supplying sucrose exogenously, Tre6P rises rapidly in parallel with sucrose, peaking at a level over 25 times higher than in C-starved seedlings (Lunn *et al.*, 2006). Tre6P levels in rosettes of soil-grown wild-type (WT) *A. thaliana* plants and the starch-deficient *pgm* mutant also vary 20-fold during the diurnal cycle, tracking diurnal changes in leaf sucrose content (Lunn *et al.*, 2006). Subsequent studies have confirmed the correlation between Tre6P and sucrose in *A. thaliana* through the diurnal cycle, during leaf senescence, and in responses to cold and altered nitrogen (N) availability (Veyres *et al.*, 2008; Wingler *et al.*, 2012; Carillo *et al.*, 2013; Nunes *et al.*, 2013*b*; Sulpice *et al.*, 2013), and also in developing potato tubers (Debast *et al.*, 2011) and wheat grains (Martínez-Barajas *et al.*, 2011). However, it is not yet clear whether Tre6P responds specifically to sucrose or is a more general signal of sugar availability.

The amount of Tre6P is determined by the relative rates of synthesis by TPS and dephosphorylation by TPP. In *A. thaliana*, there are 11 TPS or TPS-like proteins. These cluster in two phylogenetically distinct clades: class I (AtTPS1–AtTPS4) and class II (AtTPS5–AtTPS11) (Leyman *et al.*, 2001; Vogel *et al.*, 2001; Avonce *et al.*, 2006; Lunn, 2007). Only AtTPS1 has been shown unequivocally to have TPS activity, by *in vitro* assay of recombinant enzyme and by complementation of the yeast *tps1Δ* mutant, which is unable to grow on glucose-containing medium (Blázquez *et al.*, 1998; Zentella *et al.*, 1999; van Dijck *et al.*, 2002; Harthill *et al.*, 2006; Vandesteene *et al.*, 2010). The other TPS isoforms in *A. thaliana* are unable to complement the yeast *tps1Δ* mutant (Ramon *et al.*, 2009; Vandesteene *et al.*, 2010), with the possible exception of AtTPS11. Ramon *et al.* (2009) found no complementation of the yeast mutant by AtTPS11. However, Singh *et al.* (2011) reported that AtTPS11 complemented the *tps1Δ* mutant, and also the yeast *tps2Δ* mutant, which lacks TPP and is unable to grow at high temperature. However, Singh *et al.* (2011) used an inappropriate galactose-inducible promoter in their yeast complementation assays (see Vandesteene *et al.*, 2010, for further discussion), and did not confirm their results by *in vitro* assays of enzymatic activity. Each of the 10 TPP isoforms in *A. thaliana* (AtTPPA–AtTPPJ) complements the yeast *tps2Δ* mutant, indicating that they all have TPP activity (Vogel *et al.*, 1998; Vandesteene *et al.*, 2012).

*AtTPS1* is widely expressed throughout the plant. Class II *TPS* and *TPP* genes differ considerably in their spatial and temporal expression patterns, and are often restricted to localized domains (Schmid *et al.*, 2005; Ramon *et al.*, 2009; Vandesteene *et al.*, 2010, 2012). Expression of *AtTPS5* is induced by sugars in seedlings and rosettes of soil-grown plants, whereas *AtTPS8–AtTPS11* are strongly repressed by sugars (Price *et al.*, 2004; Bläsing *et al.*, 2005; Osuna *et al.*, 2007). *AtTPS9*, *AtTPS10*, *ATTPA*, *AtTPPB*, and *AtTPPJ* are repressed by N starvation and/or induced by nitrate (Wang *et al.*, 2003; Scheible *et al.*, 2004). TPS1 is potentially phosphorylated by calcium-dependent protein kinases but not by SnRK1 (Glinski and Weckwerth, 2005). TPS5–TPS7 (Moorhead *et al.*, 1999; Harthill *et al.*, 2006) and TPS8–TPS11 (Glinski and Weckwerth, 2005) are targets for phosphorylation by SnRK1 and/or calcium-dependent protein kinase. The phosphorylation status of TPS5–TPS7 is influenced by sugar availability, and the phosphorylated proteins bind 14-3-3 proteins, potentially protecting them from degradation via the ubiquitin–26S proteasome pathway (Moorhead *et al.*, 1999; Cotelle *et al.*, 2000; Harthill *et al.*, 2006). However, it is not known what role, if any, the transcriptional and post-translational regulation of the class II TPS isoforms plays in setting the level of Tre6P, as the function of these proteins remains enigmatic.

We used *A. thaliana* seedlings grown axenically in shaking liquid cultures (Scheible *et al.*, 2004) to investigate whether Tre6P is a sucrose-specific signal, and tested the effect of various inhibitors on the sucrose-induced rise in Tre6P in C-starved seedlings. Finally, we examine the relationship between Tre6P and sugars in transgenic *A. thaliana* plants that constitutively express *E. coli* TPS or TPP.

## Materials and methods

### Materials

Biochemicals were obtained from Sigma-Aldrich Chemie GmbH (http://www.sigmaaldrich.com, last accessed 21 December 2013) and enzymes from Roche Diag nostics GmbH (http://www.roche.com, last accessed 21 December 2013) unless indicated otherwise. [6,6-^2^H]Tre6P was synthesized enzymatically from [6,6-^2^H]d-glucose (Lunn *et al.*, 2006).

### *A. thaliana* seedling culture

*A. thaliana* [L.] Heynh. Columbia-0 (Col-0) seedlings were grown in axenic liquid culture for 7 d in full nutrition (FN) medium containing 15mM sucrose and then transferred to fresh FN medium (non-starved controls) or medium deficient in sucrose, nitrate, phosphate, or sulphate to induce C, N, phosphate (P) or sulphate (S) starvation, respectively (Scheible *et al.*, 2004; Nikiforova *et al.*, 2006; Morcuende *et al.*, 2007; Osuna *et al.*, 2007). Nutrient re-addition experiments were performed on 9-d-old seedlings. Inhibitors were dissolved in water or DMSO and added to C-starved seedlings 1h before sugar re-addition; for controls, an equal volume of water or DMSO was added. Seedlings were harvested under ambient illumination, washed with three changes of water, blotted, and immediately frozen in liquid nitrogen. Frozen tissue was ground using a cryogenic grinding robot (http://www.labman.co.uk, last accessed 21 December 2013) and stored at –80 °C until analysis.

### Transgenic *A. thaliana* lines

*E. coli otsA* (*TPS*) and *otsB* (*TPP*) genes were amplified by PCR (Lunn *et al.*, 2006) and inserted into the pGreen binary plasmid (Hellens *et al.*, 2000) under the control of the cauliflower mosaic virus 35S promoter. Constructs were introduced into *A. thaliana* Col-0 by *Agrobacterium*-mediated transformation using the floral dipping method (Clough and Bent, 1998). Transformants were screened using phosphinothricin (Hädrich *et al.*, 2011), and segregation analysis of progeny in the T_2_ and T_3_ generations was used to select homozygous lines containing a single transgenic locus. Expression of the heterologous proteins was confirmed by immunoblotting (Martins *et al.*, 2013). Plants were grown in a controlled environment chamber (20 °C temperature, irradiance 160 µE m^–2^ s^–1^, photoperiod of 8, 12, or 16h). Rosettes were harvested under ambient growth conditions, immediately frozen in liquid N_2_ and processed as above.

### Metabolite analysis

Tre6P was assayed in chloroform/methanol tissue extracts as described by Lunn *et al.* (2006), using an AB Sciex QTrap 5500 triple quadrupole mass spectrometer (http://www.absciex.com, last accessed 21 December 2013) which gave a lower limit of detection of 2.5fmol. Calibration was done using enzymatically verified Tre6P standards (Supplementary Methods S1 at *JXB* online). Samples were spiked with a [6,6-^2^H]Tre6P internal standard to correct for ion suppression (Lunn *et al.*, 2006).

Sucrose, glucose, fructose and starch were assayed as in Stitt *et al.* (1989). Trehalose, maltose and isomaltulose were measured in ethanolic extracts by high-performance anion-exchange chromatography with pulsed amperometric detection using a Dionex DX500 chromatograph fitted with a 2×50mm CarboPac PA1 guard-column and a 2×250mm CarboPac PA1 column in series. Hexose phosphates and UDPG were assayed enzymatically in trichloroacetic acid extracts as described by Stitt *et al.* (1989) or by LC-MS/MS in chloroform/methanol tissue extracts.

### Statistical analysis

Significance testing (Student’s *t*-test) and calculation of Pearson’s correlation coefficients was done using SigmaPlot 11 software (http://www.sigmaplot.com, last accessed 21 December 2013). Before conditional correlation analysis (Baba *et al.*, 2004) was performed, each metabolite in the experiment was standardized to mean 0 and variance 1. Conditional correlation between *X* and *Y* given a set of *n* variables (here, *n*=2) $Z=\left\{Z_{1},Z_{2},\cdots ,Z_{n}\right\}$, is the correlation between the residuals $R_{X}$ and $R_{Y}$ resulting from the linear regression of *X* with *Z* and of *Y* with *Z*. The exact Student’s *t*-test was used as the test statistic. The ‘ci.test’ function in R package ‘bnlearn’ (Scutari, 2010) was used to calculate the conditional correlation coefficient. Computations were done by considering every repeat run as an individual data point, i.e. no averaging per time point was performed prior to correlating the data.

## Results

### Response of Tre6P to sucrose, glucose, and fructose in C-starved *A. thaliana* seedlings

**Fig. 1.**
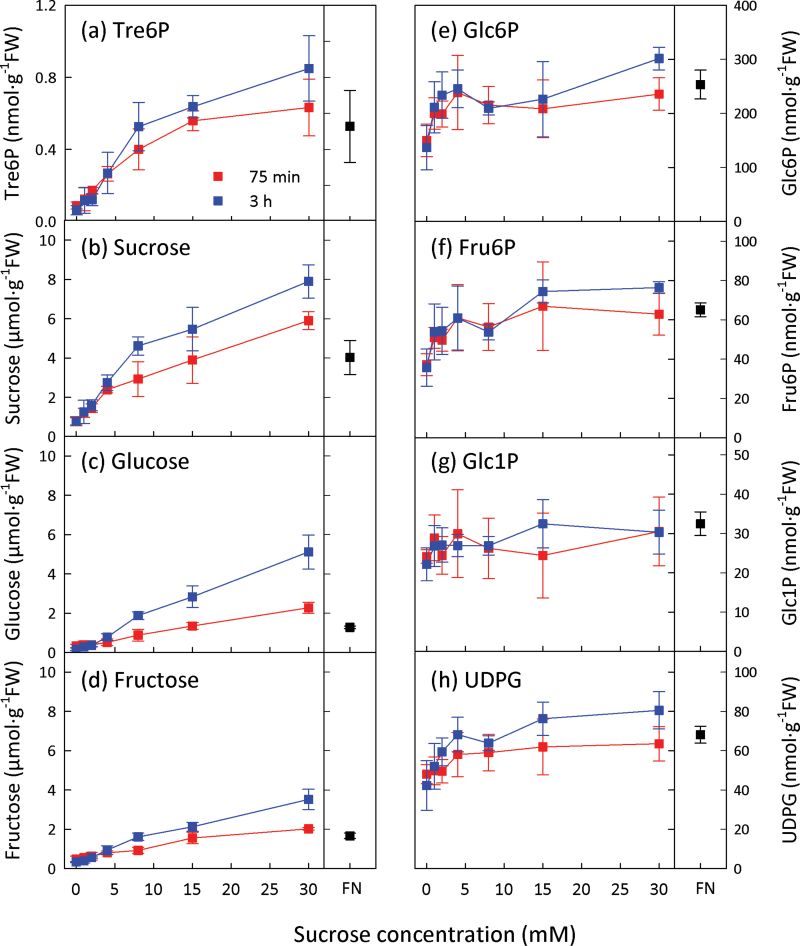
Tre6P increases after supplying sucrose to C-starved *A. thaliana* seedlings. Sucrose was supplied to C-starved 9-d-old seedlings at final concentrations of 1–30mM. Non-starved control seedlings were grown in FN medium containing 15mM sucrose. Metabolite values are means ±standard deviation (SD) (*n*=4).

C-starved seedlings were exogenously supplied with 1–30mM sucrose to assess the response range. C-starved seedlings had very low Tre6P (0.091±0.037 nmol g^–1^ FW) compared with non-starved seedlings (0.528±0.200 nmol g^–1^ FW) grown in FN medium containing 15mM sucrose. Tre6P levels increased in parallel with sucrose (Fig. 1a, b). There was a positive correlation between Tre6P and sucrose (Pearson correlation coefficient, *r*=0.937; Supplementary Fig. S1a at *JXB* online). The glucose and fructose contents of the seedlings increased progressively with the supplied sucrose concentration (Fig.1c, d). Hexose phosphates and UDPG rose about 2-fold as the exogenous concentration of sucrose increased up to 4mM, with little or no further increase at higher sucrose concentrations (Fig.1e–h). Tre6P correlated with glucose (*r*=0.846) and fructose (*r*=0.886) but less well with Glc6P (*r*=0.499) and UDPG (*r*=0.640) (Supplementary Fig. S1b–e).

**Fig. 2.**
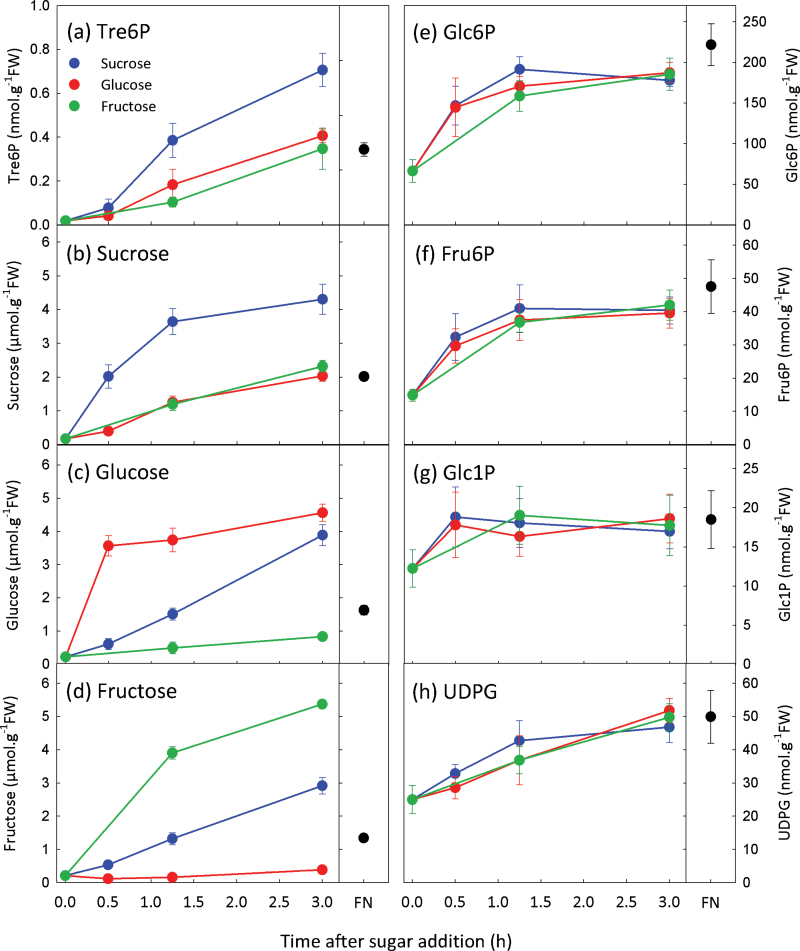
Exogenously supplied hexoses increase both the sucrose and Tre6P content of C-starved *A. thaliana* seedlings. Sucrose (final concentration 15mM), glucose (30mM), or (fructose (30mM) was supplied to C-starved 9-d-old seedlings. Non-starved control samples were grown in FN medium containing 15mM sucrose. Metabolite values are means ±SD (*n*=4).

To investigate the specificity of the response, hexose-equivalent concentrations of sucrose (15mM), glucose (30mM), or fructose (30mM) were supplied to C-starved seedlings (Fig. 2). Tre6P increased almost 40-fold after sucrose feeding, reaching levels well above those found in non-starved seedlings, but rose more slowly when seedlings were supplied with glucose or fructose. Sucrose increased rapidly after sucrose feeding, while glucose and fructose rose more slowly. Conversely, when supplied with glucose or fructose, sucrose levels increased more slowly than the hexose supplied. Hexose phosphates and UDPG behaved in a similar manner whichever sugar was provided, increasing about 2-fold to reach the levels found in non-starved seedlings. Overall, Tre6P was highly correlated with sucrose (*r*=0.768), and less strongly correlated with glucose (*r*=0.457), fructose (*r*=0.363), Glc6P (*r*=0.522), and UDPG (*r*=0.697) (Supplementary Fig. S2 at *JXB* online).

The experiments in Figs 1 and 2 confirmed that Tre6P rises after supplying sucrose to C-starved seedlings (Lunn *et al.*, 2006) but also showed significant responses of Tre6P to glucose and fructose feeding. Hexose sugars increased after sucrose feeding, and vice versa. To deconvolute the results, we performed conditional correlation analysis (Baba *et al.*, 2004) treating the sucrose, glucose, and fructose addition series from the second experiment as separate datasets. The conditional correlation between sucrose and Tre6P was highly significant in the first experiment (*P*=2.7×10^–9^), significant for the sucrose addition series (*P*=0.04), and highly significant for the glucose addition series (*P* =6.9×10^–6^) (Table 1). Although the conditional correlation between Tre6P and sucrose for the fructose addition series was not statistically significant (*P*=0.09), it was higher than those between Tre6P and the hexose sugars (Table 1).

**Table 1.** Conditional correlation between Tre6P, sucrose, glucose, and fructoseMetabolite data were taken from the experiments presented in Figs 1 and 2. The value in the parentheses is the corresponding *P* value. Significant conditional correlations (*P*<0.05) are indicated in bold.

Addition	Conditional correlation coefficient (*P* value)		
	Tre6P-glucose	Tre6P-fructose	Tre6P-sucrose
**Experiment 1**			
Sucrose	–0.17 (0.18)	0.11 (0.39)	**0.67** (2.7×10^–9^)
**Experiment 2**			
Sucrose	0.20 (0.42)	0.07 (0.78)	**0.48** (0.04)
Glucose	–0.18 (0.48)	–0.45 (0.06)	**0.85** (6.9×10^–6^)
Fructose	0.01 (0.96)	–0.03 (0.92)	0.49 (0.09)

### Response of Tre6P to trehalose and sugar analogues

**Fig. 3.**
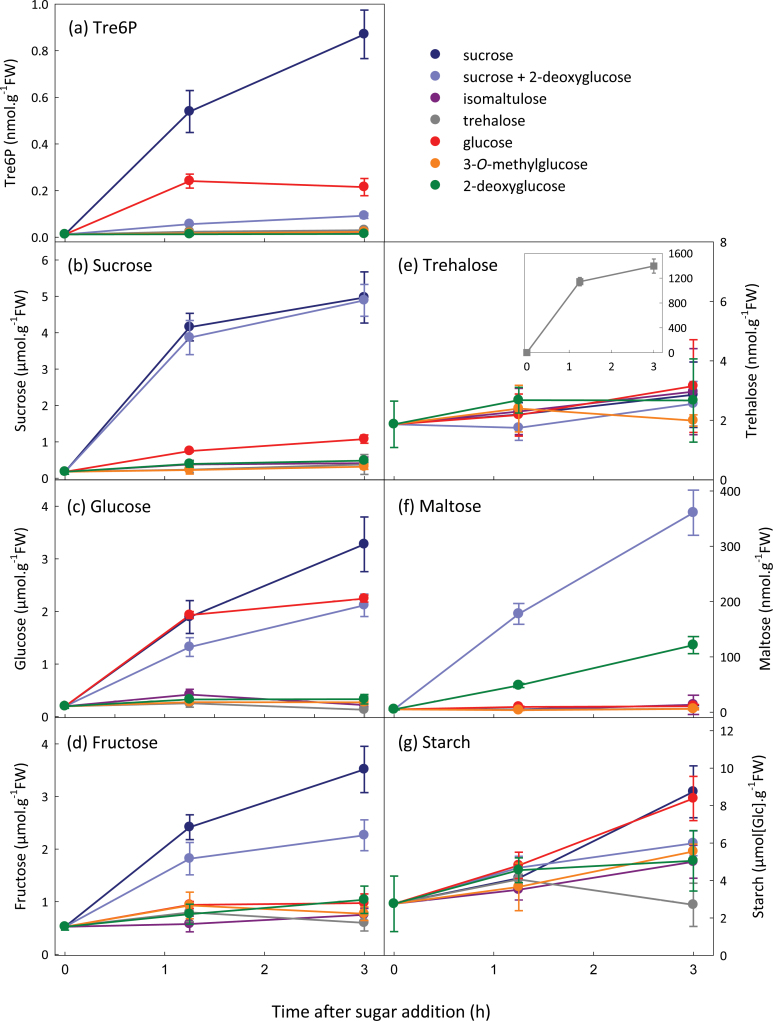
Effect of exogenous sugars and sugar analogues on Tre6P content of C-starved *A. thaliana* seedlings. Sucrose (±2-DOG), isomaltulose, trehalose, glucose, 3-OMG, or 2-DOG (all at final concentrations of 15mM) were supplied to C-starved 9-d-old seedlings. Metabolite values are means ±SD (*n*=4).

To investigate further the specificity of the Tre6P response to sugar feeding, C-starved seedlings were supplied with trehalose, the sucrose analogue isomaltulose, a glucose analogue that is taken up but not phosphorylated (3-*O*-methylglucose; 3-OMG), and a glucose analogue that is taken up and phosphorylated by hexokinase (2-deoxyglucose; 2-DOG). Sucrose triggered a larger increase in Tre6P than glucose feeding, matching the corresponding changes in sucrose (Fig. 3). Trehalose and isomaltulose had no effect on Tre6P levels. Trehalose and isomaltulose were readily taken up the seedlings but appeared to be metabolized very slowly as there was no increase in sucrose, glucose, fructose (Fig. 3), or hexose phosphates (data not shown). Supplying 3-OMG or 2-DOG had no effect on Tre6P or sucrose levels. The 2-DOG-treated seedlings contained very low levels of hexose phosphates and UDPG (Supplementary Fig. S3 at *JXB* online). When sucrose and 2-DOG were supplied together, sucrose increased to the same extent as when sucrose was supplied alone, but there was only a small rise in Tre6P. Glucose and fructose rose after supplying sucrose + 2DOG (Fig. 3), but hexose phosphates and UDPG decreased or were unchanged (Supplementary Fig. S3).

Tre6P was more highly correlated with glucose (*r*=0.729) and fructose (*r*=0.818) than with sucrose (*r*=0.671) when all of the samples were included (Supplementary Fig. S4 at *JXB* online). When the sucrose+2-DOG treatment was excluded, Tre6P correlated more strongly with sucrose (*r*=0.946) than with glucose (*r*=0.804) or fructose (*r*=0.932) (Supplementary Fig. S4; see values in parentheses).

### Does the response of Tre6P to hexose sugars depend on their conversion to sucrose?

**Fig. 4.**
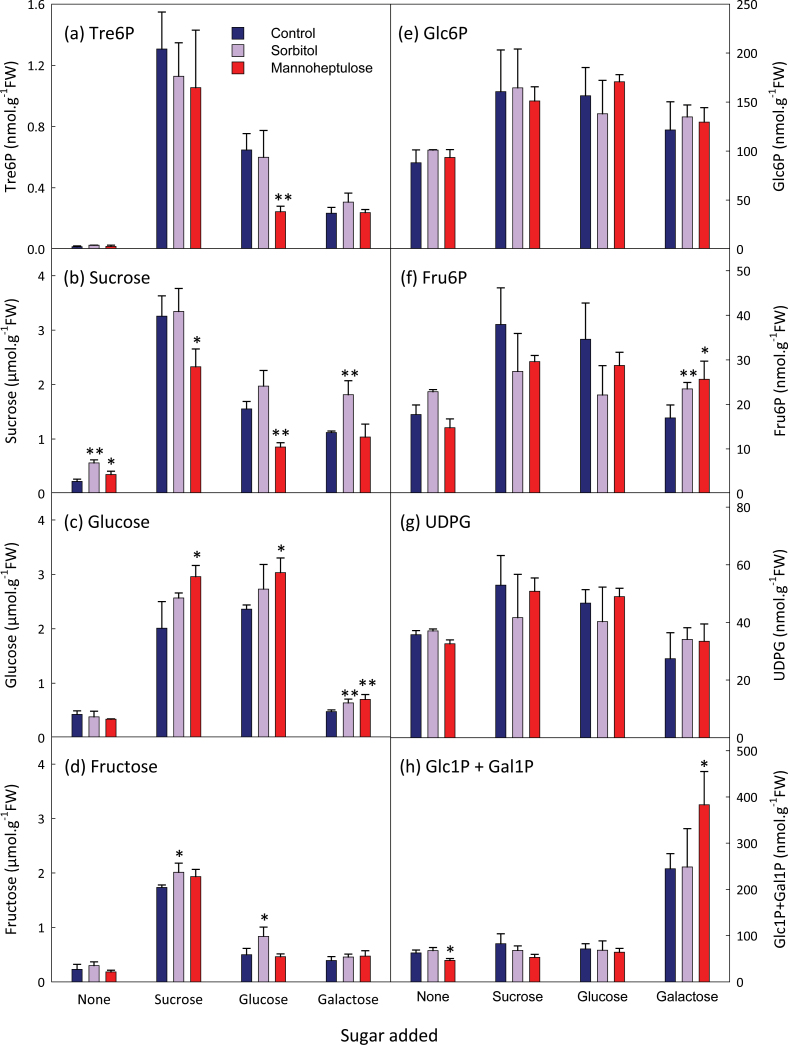
Effect of mannoheptulose on sucrose and hexose-induced increases in the Tre6P content of *A. thaliana* seedlings. After 7 d growth in 30ml of FN medium (15mM sucrose), seedlings were transferred to 15ml of sucrose-free medium on day 8 and again on day 9. The C-starved seedlings were incubated with 100mM mannoheptulose or 100mM sorbitol (osmotic control) for 1h before supplying sucrose (final concentration 7.5mM), glucose (15mM), or galactose (15mM). Metabolite values are mean±SD (*n*=2–4). Asterisks indicate significant differences (Student’s *t*-test) between the sorbitol- or mannoheptulose-treated seedlings and the respective controls: *P<0.05; **P<0.01. Glc1P, glucose 1-phosphate; Gal1P, galactose 1-phosphate.

Mannoheptulose is a weak competitive inhibitor of hexokinase (*K*_i_=0.5–20mM; Claeyssen and Rivoal, 2007). When supplied with sucrose, neither 100mM mannoheptulose nor 100mM sorbitol (osmotic control) had any effect on the rise in Tre6P (Fig. 4). Mannoheptulose decreased the rise in Tre6P and sucrose when seedlings were fed with glucose, but hexose phosphates and UDPG were not significantly affected by mannoheptulose.

We also investigated the response of Tre6P to galactose, which is phosphorylated to galactose 1-phosphate by galactokinase rather than hexokinase. There was a moderate increase in Tre6P after galactose feeding, which was not inhibited by mannoheptulose (Fig. 4). Sucrose levels were similar in galactose and glucose-fed seedlings. Glc6P, fructose 6-phosphate (Fru6P) and UDPG levels in the galactose-fed seedlings were similar to or slightly higher than in C-starved seedlings.

In this experiment, Tre6P was highly correlated with sucrose (*r*=0.918) and fructose (*r*=0.886), but less so with glucose (*r*=0.661), Glc6P (*r*=0.643), or UDPG (*r*=0.567) (Supplementary Fig. S5 at *JXB* online).

### Response of Tre6P to N, P, and S availability

Seedling cultures were also used to investigate whether Tre6P responds to changes in N, P, or S status. N-starved seedlings had over three times the level of Tre6P in non-starved seedlings (Supplementary Table S1 at *JXB* online). Tre6P was unaffected by KCl addition, showed a slight non-significant decrease 3h after supplying KNO_3_, and decreased 35–40% after supplying NH_4_Cl. There were parallel changes in the sucrose content of the seedlings. There were only small, non-significant changes in Tre6P content in response to P starvation and P feeding (Supplementary Table S1). It has been reported previously from identical experiments that changes in P status have only a small impact on sucrose levels in seedlings (Morcuende *et al.*, 2007). There was no difference in Tre6P content between S-starved and non-starved seedlings (Supplementary Table S1). Tre6P fell slightly after supplying K_2_SO_4_, but the changes were small (<20%) and not significant.

Tre6P correlated with sucrose (*r*=0.892 and 0.653 in the N and S feeding experiments, respectively; Supplementary Fig. S6a, d, at *JXB* online). Tre6P correlated with glucose and fructose in the N feeding experiment (*r*=0.817 and 0.829, respectively), but only very weakly in the S feeding experiment (*r*=0.308 and 0.215, respectively) (Supplementary Fig. S6e, f).

### Effect of TPS or TPP overexpression on Tre6P and sugars

We generated transgenic lines that constitutively expressed the *E. coli otsA* (35S::TPS) or *otsB* (35S::TPP) genes, with the aim of perturbing the network linking Tre6P to sucrose. Our lines showed similar visual phenotypes to those reported by Schluepmann *et al.* (2003) (Supplementary Figs S7 and 8, and Supplementary Methods 3, at *JXB* online).

Plants were grown in 8, 12, or 16h photoperiods and harvested at the end of the day (ED) and the end of the night (EN). Tre6P was 4- to 11-fold higher in 35S::TPS than in WT, with all differences being highly significant (*P<*0.001), whereas the Tre6P content of 35S::TPP was not significantly different from WT Col-0 (Supplementary Fig. S9 at *JXB* online). Sucrose levels were consistently lower in 35S::TPS under all conditions (*P*<0.05), and increased significantly in 35S::TPP with 12 and 16h daylengths (*P*<0.05). There were much smaller and less consistent differences in glucose, while fructose showed a similar trend to sucrose but with fewer significant differences between the genotypes.

**Fig. 5.**
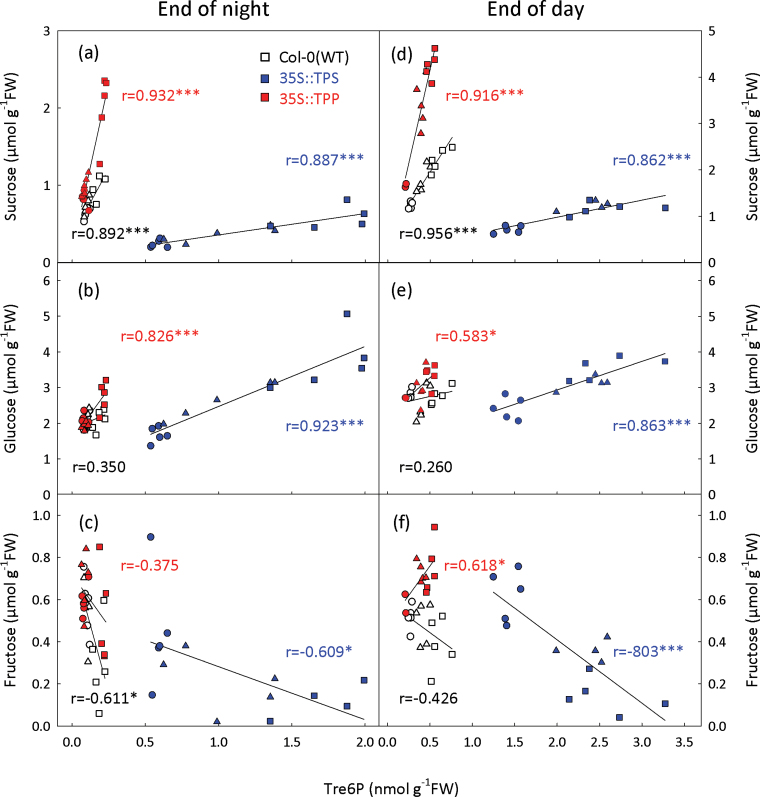
Effect of constitutive TPS and TPP expression on the relationship of Tre6P to sugars in *A. thaliana*. WT (Col-0), 35S::TPS, and 35S::TPP plants were grown under 8h (circles), 12h (triangles), or 16h (squares) day lengths. Rosettes were harvested from 25-d-old plants at (a–c) the end of the night and (d–f) end of the day. The Tre6P content of the individual samples was plotted against: sucrose (a, d), glucose (b, e), and fructose (c, f). The Pearson correlation coefficient (*r*) for each metabolite pair is shown. Asterisks indicate significant correlations: *P<0.05, ***P<0.001.

Tre6P was highly (*P*<10^–4^) and positively correlated with sucrose in WT Col-0, 35S::TPS 35S::TPP, at both ED and EN (Fig. 5). The Tre6P:sucrose ratio showed a highly significant separation of the genotypes (Table 2). The correlation between Tre6P and glucose or fructose was generally weaker. Tre6P was positively correlated with glucose (*P*<10^–4^) at ED and EN in the 35S::TPS plants, at EN in the 35S::TPP plants (*P*<0.001) but not in WT Col-0 at either time point (Fig. 5). Tre6P was negatively correlated with fructose (*P*<0.05) at the ED and EN in the 35S::TPS plants, at EN in WT Col-0 (*P*<0.05) and positively correlated with fructose in at ED in the 35S::TPP plants (*P*<0.05) (Fig. 5).

**Table 2.** *Tre6P:sucrose ratios in WT Col-0, 35S::TPS and 35S::TPP plants*Ratios are means ±SD. The *P* value (Student’s *t*-test) refers to differences versus WT (Col-0) plants grown and harvested under the same conditions. Reference indicates the data source and number of samples (*n*). EN, end of night; ED, end of day; NA, not applicable.

Genotype	Tre6P:sucrose (nmol µmol^–1^)	*P* value (vs WT)	Reference
WT (Col-0)^a^	0.11±0.04	NA	Fig. 1 (*n*=63)
WT (Col-0)^a^	0.12±0.05	NA	Fig. 2 (*n*=40)
WT (Col-0)^a^	0.11±0.09	NA	Fig. 3 (*n*=50)
WT (Col-0)^a^	0.25±0.14	NA	Fig. 4 (*n*=37)
WT (Col-0)^a^ (N series)	0.18±0.05	NA	Fig. S6a (*n*=37)
WT (Col-0)^a^ (S series)	0.12±0.03	NA	Fig. S6d (*n*=54)
WT (Col-0)^a^	0.14±0.05	NA	Fig. 7 (*n*=36)
WT (Col-0)^b^ (EN)	0.16±0.03	NA	Fig. 5 (*n*=15)
WT (Col-0)^b^ (ED)	0.24±0.03	NA	Fig. 5 (*n*=15)
35S::TPS^b^ (EN)	2.84±0.61	2.4×10^–16^	Fig. 5 (*n*=15)
35S::TPS^b^ (ED)	2.07±0.27	1.4×10^–20^	Fig. 5 (*n*=14)
35S::TPP^b^ (EN)	0.10±0.02	2.3×10^–6^	Fig. 5 (*n*=15)
35S::TPP^b^ (ED)	0.12±0.01	8.3×10^–13^	Fig. 5 (*n*=12)

^*a*^ Seedling culture.

^*b*^ Rosettes from soil-grown plants.

In summary, constitutive expression of a heterologous TPS increased the Tre6P:sucrose ratio with respect to WT plants, due to both higher Tre6P and lower sucrose. TPP expression decreased the Tre6P:sucrose ratio due almost entirely to an increase in sucrose. However, in the individual genotypes, the ratio was maintained within a narrow range across the three growth conditions.

### Transcriptional and translational regulation of the Tre6P response to sucrose

We investigated the effect of transcriptional and translational inhibitors on the Tre6P response to sucrose. In a preliminary experiment, we found that 0.6mM cordycepin was more effective than 20 µM α-amanitin at inhibiting induction of five sugar-inducible genes by sucrose (Supplementary Table S2 at *JXB* online). Cycloheximide was used to inhibit protein synthesis.

**Fig. 6.**
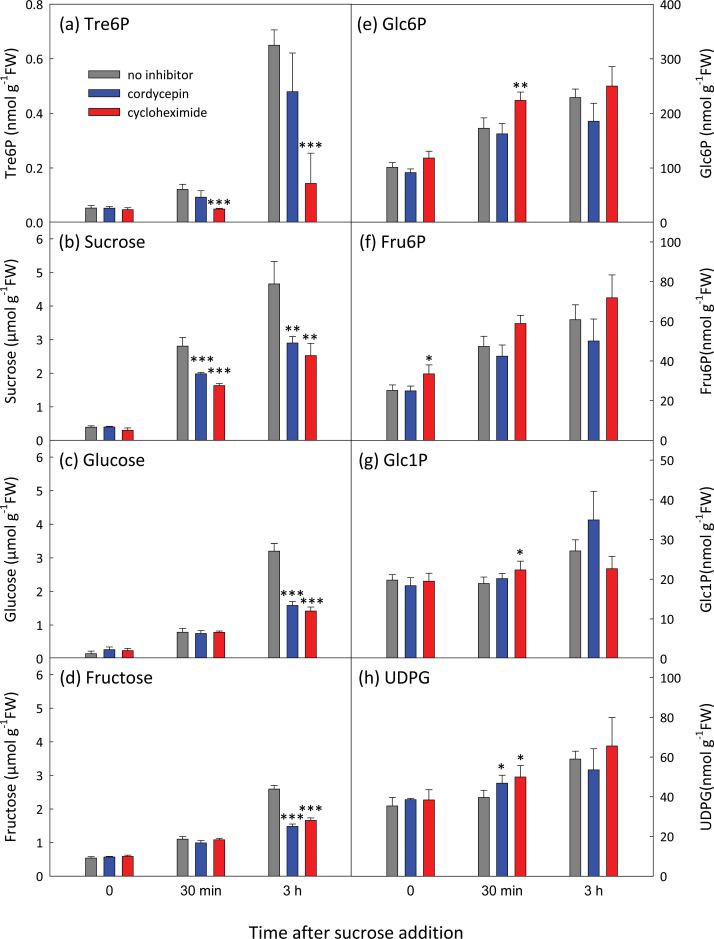
Effect of cordycepin and cycloheximide on sucrose-induced changes in the Tre6P content of *A. thaliana* seedlings. C-starved 9-d-old seedlings were incubated with 0.6mM cordycepin or 100 µM cycloheximide or without inhibitor for 1h before addition of sucrose (final concentration 15mM). Metabolite values are means ±SD (*n*=4). Asterisks indicate significant differences (Student’s *t*-test) between the cordycepin- or cycloheximide-treated seedlings and the controls: *P<0.05; **P<0.01; ***P<0.001.

Treatment with cordycepin led to a small and non-significant attenuation of the sucrose-induced rise in Tre6P (Fig. 6). In contrast, cycloheximide almost abolished the response. The sucrose, glucose, and fructose contents of the cordycepin and cycloheximide-treated seedlings were about half those of the no-inhibitor controls. Cordycepin generally had little effect on hexose phosphate and UDPG levels. These intermediates tended to be higher in the cycloheximide-treated seedlings than in the controls, but the differences were mostly small.

We measured the abundances of *TPS*, *TPP*, and *TRE1* transcripts by qRT-PCR (see Supplementary Methods S2, with results shown in Supplementary Fig. S10; available in *JXB* online). Sucrose feeding led to a 7-fold induction of *TPS5*, with smaller increases in *TPS1*, *TPS4*, and several *TPP* transcripts, a large decrease in *TPS8–TPS11* transcripts (>50-fold for *TPS8*), and smaller falls in *TPS6*, *TPS7*, and several *TPP* transcripts and *TRE1*. Transcript levels were generally lower in seedlings treated with cordycepin, including the sucrose-inducible *TPS5* gene. A broadly similar trend was observed in seedlings treated with cordycepin and then supplied with sucrose, although *TPS8*, *TPS9*, and *TPS10* transcript levels were noticeably lower than in seedlings treated with cordycepin alone.

The observed inhibition by cycloheximide suggested that protein synthesis is necessary for the Tre6P response to sucrose. We performed polysome loading analysis (Piques *et al.*, 2009; see Supplementary Methods S2) to investigate whether sucrose feeding affected translation of *TPS*, *TPP*, or *TRE1* mRNAs. In C-starved seedlings, only 31% of the ribosomes were in the polysomal fraction, rising to 42% within 30min of feeding sucrose and 59% by 3h. No significant changes in the ribosomal occupancy of the *TPS*, *TPP*, or *TRE1* mRNAs were observed after sucrose feeding, except for a 20% decrease in the polysome loading of *TPPJ* (Supplementary Table S3 at *JXB* online). Immunoblotting of seedling extracts indicated that sucrose feeding had little effect on the abundance or size of the TPS1 protein, even in cycloheximide-treated seedlings in which the Tre6P response to sucrose was almost abolished (Supplementary Fig. S11 at *JXB* online).

### Post-translational regulation of the Tre6P response to sucrose

We incubated C-starved seedlings with protein kinase or protein phosphatase inhibitors for 1h before supplying sucrose (Supplementary Fig. S12 at *JXB* online). The broad-specificity protein kinase inhibitors staurosporine and K252a strongly inhibited the sucrose-induced rise in Tre6P. Staurosporine had no effect on the level of sucrose compared with the controls, but sucrose was significantly higher in the K252a-treated seedlings. Okadaic acid (a protein phosphatase type 2A inhibitor) and calyculin A (a protein phosphatase type 1 inhibitor) also strongly inhibited the response of Tre6P to sucrose feeding. Seedlings treated with these inhibitors also had lower levels of sucrose than the controls. Glucose and fructose were decreased in all of the treatments. Glc6P and Fru6P were lower in seedlings treated with the protein kinase inhibitors.

**Fig. 7.**
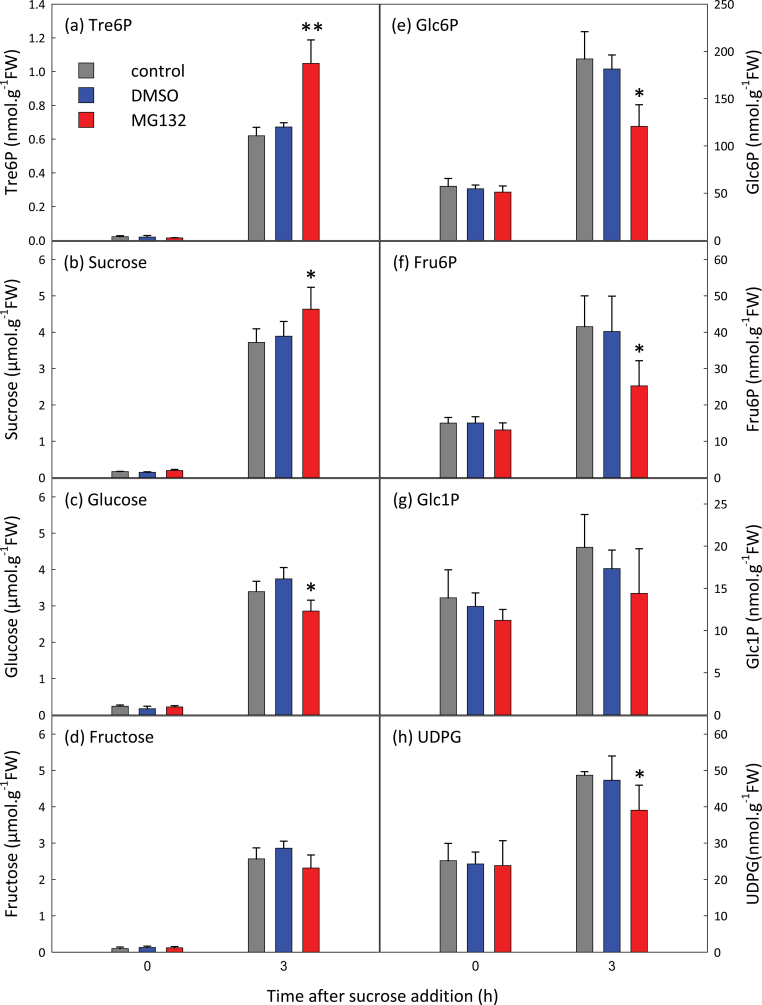
Effect of MG132 on sucrose-induced changes in the Tre6P content of *A. thaliana* seedlings. C-starved 9-d-old seedlings were incubated with 34mM DMSO or 100 µM MG132+34mM DMSO for 1h before supplying sucrose (final concentration 15mM). Metabolite values are means ±SD (*n*=4). Asterisks indicate significant differences (Student’s *t*-test) between the DMSO- or MG132+DMSO-treated seedlings and the controls: *P<0.05; **P<0.01.

We investigated the effect of MG132, an inhibitor of the 26S proteasomal protease. Tre6P rose to higher levels in sucrose-fed seedlings treated with MG132 than in the controls (Fig. 7). MG132-treated seedlings had more sucrose but slightly less glucose and hexose phosphates than the controls. Tre6P was more highly correlated with sucrose (*r*=0.952) than with glucose (*r*=0.862) or fructose (*r*=0.875) (Supplementary Fig. S13 at *JXB* online). The Tre6P:sucrose ratio was similar in MG132-treated and control seedlings.

## Discussion

### Is Tre6P a sucrose-specific signal?

It is well established that Tre6P levels in plant tissues change in parallel with endogenous or imposed fluctuations in sucrose content. This relationship has been observed in *A. thaliana* seedlings (Lunn *et al.*, 2006; Nunes *et al.*, 2013*b*), rosettes (Lunn *et al.*, 2006; Veyres *et al.*, 2008; Wingler *et al.*, 2012; Carillo *et al.*, 2013; Sulpice *et al.*, 2013), and shoot apices (Wahl *et al.*, 2013), as well as in developing potato tubers (Debast *et al.*, 2011) and wheat grains (Martínez-Barajas *et al.*, 2011). Tre6P was less obviously linked to sucrose during grape berry development (Dai *et al.*, 2013), but any link might easily be hidden by the very high sugar concentrations accumulated in the large vacuoles of grape parenchyma cells.

In all of our experiments with *A. thaliana* seedling cultures, Tre6P was highly correlated with sucrose, irrespective of the type of sugar supplied to C-starved seedlings. Furthermore, Tre6P correlated more strongly with sucrose than with any of the other metabolites measured in our study, with two exceptions. One was seedlings supplied with sucrose and 2-DOG (Supplementary Fig. S4), in which the UDPG and hexose phosphate pools were greatly depleted (Supplementary Fig. S3), presumably due to sequestration of P_i_ in 2-deoxyglucose 6-phosphate. The resulting depletion of phosphorylated metabolites might restrict the capacity of the cells to synthesize Tre6P. The other exception was in the N starvation experiment, where there was a slightly higher correlation between Tre6P and fructose than sucrose, but this was driven by a single outlier (Supplementary Fig. S6). Tre6P correlated strongly with sucrose in rosettes of WT, 35S::TPS, and 35S::TPP plants, but more weakly or even negatively with glucose and fructose (Fig. 5).

Tre6P levels increased in seedlings after feeding glucose, fructose, or galactose, but not as strongly or as rapidly as in the response to sucrose (Figs 2–4). Sucrose levels rose after supplying hexose sugars, and Tre6P reflected these changes in sucrose content more closely than it did the accumulation of hexose sugars. Conditional correlation analysis indicated that the correlation between sucrose and Tre6P, excluding the influence of glucose and fructose, was significant or highly significant (Table 1). In contrast, the correlations between Tre6P and glucose or fructose, excluding the influence of sucrose, were not significant. These results suggested that sucrose has a direct impact on Tre6P levels, whereas the influence of glucose and fructose on Tre6P is both indirect and dependent on sucrose.

Feeding of other sugars corroborated this conclusion. Tre6P did not increase when seedlings were supplied with 3-OMG or 2-DOG (Fig. 3), two analogues of glucose that are taken up by plant cells but not metabolized to sucrose (Jang and Sheen, 1994). Trehalose and isomaltulose, an isomer of sucrose that has been used to investigate sugar signalling pathways (Sinha *et al.*, 2002), were readily taken up by the seedlings but had no significant impact on Tre6P levels (Fig. 3). Mannoheptulose, an inhibitor of hexokinase, restricted the rise in both sucrose and Tre6P after feeding glucose (Fig. 4). The observation that mannoheptulose did not prevent replenishment of the UDPG and hexose phosphate pools after glucose feeding suggests that it also inhibits some other step in the biosynthesis of sucrose. Whatever the mechanism by which mannoheptulose affected sucrose levels, Tre6P was highly correlated with sucrose (*r*=0.918) across these treatments (Supplementary Fig. S5a).

Our glucose-feeding experiments differed from those of Nunes *et al.* (2013*b*), who monitored the effects of sugar feeding for up to 3 d after supplying glucose but observed no significant rise in Tre6P, even though sucrose levels increased about 2-fold. We have no explanation for this difference, but note that in our hands the effect of hexose feeding on Tre6P was highly reproducible, being observed in six other independent experiments in addition to the three experiments presented in Figs 2–4. We would argue that our shorter-term feeding experiments reveal more about the early responses to sugars, and so give greater insight into which sugars trigger changes in the level of Tre6P.

We observed no significant increase in Tre6P after supplying 15mM trehalose to C-starved seedlings, even though trehalose was taken up (Fig. 3). *A. thaliana* seedlings grown on medium containing 100mM trehalose show severely restricted growth, which has been attributed to high levels of Tre6P arising from inhibition of TPP by trehalose (Schluepmann *et al.*, 2004; Delatte *et al.*, 2011). If direct inhibition of TPP were the primary cause of elevated Tre6P levels in trehalose-grown seedlings, we might have expected to see a rapid response of Tre6P to trehalose feeding. However, Tre6P levels were not significantly altered, even 3h after supplying trehalose (Fig. 3). This suggests that direct inhibition of TPP is not the only mechanism by which exogenous trehalose affects Tre6P levels when seedlings are grown long term on trehalose-containing medium, complicating interpretation of their phenotypes.

In conclusion, our results provide new lines of evidence that: (i) Tre6P acts as a signal metabolite for sucrose status in plants; (ii) the responses of Tre6P to hexoses and other sugars are indirect and dependent on changes in sucrose; and (iii) other nutrients that are essential for growth – N, P, and S – have relatively little influence on Tre6P except via their effects on sucrose levels.

### Regulation of Tre6P content by sucrose

In addition to its central role in plant metabolism, sucrose is a key regulator of many processes (Lunn and MacRae, 2003; Lunn, 2008). Sucrose transcriptionally regulates thousands of genes (Bläsing *et al.*, 2005; Osuna *et al.*, 2007), exerts global and protein-specific control over translation (Hummel *et al.*, 2009; Rahmani *et al.*, 2009; Pal *et al.*, 2013), and affects the activity and stability of many proteins, either directly or via its influence on post-translational modifications (Moorhead *et al.*, 1999; Cotelle *et al.*, 2000; Harthill *et al.*, 2006). Thus, there is potential for sucrose to regulate the level of Tre6P in many different ways.

Our experiments showed that *de novo* transcription is not required for the Tre6P response to sucrose. Inhibition of transcription by cordycepin had only a small impact on the Tre6P response to sucrose in seedlings, which could be accounted for by a slightly lower accumulation of sucrose (Fig. 6). Thus, even though *TPS1* and *TPS5* transcripts are induced by sucrose (Supplementary Fig. S10), this cannot be an essential factor in the sucrose-dependent increase in Tre6P. Interestingly, cordycepin led to a fall in abundance of several of the *TPS* and *TPP* transcripts that, in the case of *TPS8*, *TPS9*, and *TPS10*, was even more marked when sucrose was supplied together with cordycepin. This indicates that these transcripts are rapidly turned over in C-starved seedlings and are further destabilized by sucrose. It is unclear if this contributes to the sucrose-dependent rise in Tre6P, as the functions of TPS8–TPS10 are unknown. Studying the sucrose response in null mutants of these genes might be informative, but given their close phylogenetic relationship and likely redundancy (Lunn, 2007), it would probably be necessary to generate a *tps8 tps9 tps10* triple mutant in order to observe any difference.

*De novo* protein synthesis is necessary for sucrose to exert its effect on Tre6P levels. Cycloheximide almost completely blocks the increase in Tre6P, even though sucrose levels resembled those after supplying cordycepin (Fig. 6). This effect was observed in three independent experiments (see also Supplementary Fig. S11, and data not shown). We do not yet know which protein(s) needs to be synthesized in order for Tre6P levels to rise after sucrose feeding. Polysome loading indicated that none of the TPS isoforms was translationally regulated by sucrose (Supplementary Table S3). Immunoblotting confirmed that there was no obvious increase in protein abundance of TPS1 (Supplementary Fig. S11a), so maximal catalytic activity of TPS is unlikely to be increased. Among the TPP isoforms, only TPPJ appeared to be regulated translationally by sucrose, although the effect was small and of doubtful importance given the multiplicity of catalytically active TPP isoforms that were unaffected. From these results, we conclude that some other protein(s) must be synthesized in order for Tre6P levels to rise in response to sucrose feeding. The bZIP11 transcription factor is regulated translationally by sucrose and implicated in transcriptional regulation of trehalose metabolism (Rahmani *et al.*, 2009; Ma *et al.*, 2011). However, our cordycepin experiments suggested that transcriptional regulation makes little, if any, contribution to the Tre6P response to sucrose in seedlings, arguing against a role for bZIP11 in this short-term response.

Direct metabolic activation of TPS activity or inhibition of TPP by sucrose cannot be ruled out as contributing factors in the Tre6P response to sucrose. Indeed, very little is known about the kinetic properties of the plant enzymes. Although cytosolic concentrations of sucrose can exceed 100mM, the activity of *Selaginella lepidophylla* TPS1 is not affected by up to 400mM sucrose (Valenzuela-Soto *et al.*, 2004). TPP is structurally related to sucrose-phosphate phosphatase. *Synechocystis* sp. PCC6803 sucrose-phosphate phosphatase is competitively inhibited by trehalose (*K*_i_=26mM) and sucrose (*K*_i_=161mM) (Fieulaine *et al.*, 2007), suggesting that TPP might also be inhibited by these disaccharides. The sucrose-induced rise in the substrates of TPS, UDPG and Glc6P, also has potential to stimulate the synthesis of Tre6P, and their availability is clearly necessary for the response, as shown by the inhibitory effect of 2-DOG (Fig. 3 and Supplementary Fig. S3). However, the correlation between Tre6P and UDPG or Glc6P was weaker than with sucrose (Supplementary Figs S1, S2, and S5), indicating that sucrose acts independently of changes in these metabolites. It is also worth noting that direct regulation of TPS and/or TPP by sucrose and substrate-driven effects on TPS activity cannot easily explain the cycloheximide sensitivity of the Tre6P response to sucrose. Thus, we conclude that direct biochemical effects of sucrose might contribute to the response, but other mechanisms predominate.

The sensitivity of the sucrose-induced rise in Tre6P to the protein kinase and phosphatase inhibitors must be interpreted cautiously. Staurosporine and K252a-treated seedlings had similar or even elevated sucrose levels compared with controls but low Tre6P (Supplementary Fig. S12). Both inhibitors target a broad spectrum of protein kinases, so they probably disrupt many cellular processes including protein synthesis (Redpath and Proud, 1989), which, like treatment with cycloheximide, would be expected to block the rise in Tre6P. The lower Tre6P levels in seedlings treated with okadaic acid and calyculin A might simply reflect the low levels of sucrose in these treatments. While there is obvious potential for phosphorylation of various TPS isoforms to play a role (Moorhead *et al.*, 1999; Glinski and Weckwerth, 2005; Harthill *et al.*, 2006), further experiments will be needed to understand how the protein kinase inhibitors affect the Tre6P response to sucrose.

Seedlings treated with MG132, which inhibits the 26S proteasomal protease, had more Tre6P than the no-inhibitor controls (Fig. 7). However, this might be attributable to their higher sucrose content, rather than any enhancement of the response mechanism. The MG132-treated seedlings had reduced amounts of glucose, hexose phosphates, and UDPG, suggesting that MG132 partially inhibited sucrose catabolism allowing greater accumulation of sucrose, and hence Tre6P. TPS1 contains an autoinhibitory domain at the N terminus, whose removal considerably increases the enzyme’s activity (van Dijck *et al.*, 2002). However, we found no evidence that the TPS1 protein is truncated *in vivo* after sucrose feeding (Supplementary Fig. S11a).

In conclusion, our inhibitor experiments revealed that *de novo* protein synthesis is essential for the response of Tre6P to sucrose. Sucrose does not appear to regulate translation of the TPS and TPP proteins themselves (Supplementary Table S3), suggesting that new regulatory proteins need to be synthesized. Potential candidates include regulatory subunits of heteromeric TPS/TPP enzyme complexes as found in yeast (Bell *et al.*, 1998), or protein kinases that modulate the activity of TPS or TPP by protein phosphorylation.

### The Tre6P–sucrose nexus

The *E. coli* TPS or TPP enzymes have a much simpler structure than their counterparts from plants (Lunn, 2007) and have no known regulatory properties. Therefore, it was anticipated that constitutive expression of these bacterial enzymes would override the regulatory mechanisms controlling the activity of the endogenous plant TPS and TPP, thereby breaking the tight connection between Tre6P and sucrose. Unexpectedly, we found that the strong correlation between Tre6P and sucrose seen in WT Col-0 plants was maintained in the 35S::TPS and 35S::TPP lines (Fig. 5), in which Tre6P levels were manipulated in different ways. However, the Tre6P:sucrose ratio was shifted to a higher or lower level, respectively (Table 2).

Schluepmann *et al.* (2003) reported Tre6P levels of 12 nmol g^–1^ FW in 35S::TPS plants and 0.5–1.0 nmol g^–1^ FW in 35S::TPP, compared with 5 nmol g^–1^ FW in WT plants. Our 35S::TPS plants also had more Tre6P than the WT plants. However, there was no significant difference from WT in the Tre6P content of our 35S::TPP plants, although they showed the morphological features typical of constitutive TPP overexpressing lines (Supplementary Figs S7 and 8). This differs from the 35S::TPP plants analysed by Schluepmann *et al.* (2003), which were reported to have 5 to 10 times less Tre6P than WT plants. However, their measurements were made using a yeast hexokinase inhibition assay, which is less sensitive and less specific than the LC-MS/MS-based method and prone to interference by other components in plant extracts (see Lunn *et al.*, 2006, for further discussion). In agreement with our findings, Wingler *et al.* (2012) found no significant differences between WT and 35S::TPP plants in their Tre6P content when measured by LC-MS/MS.

**Fig. 8.**
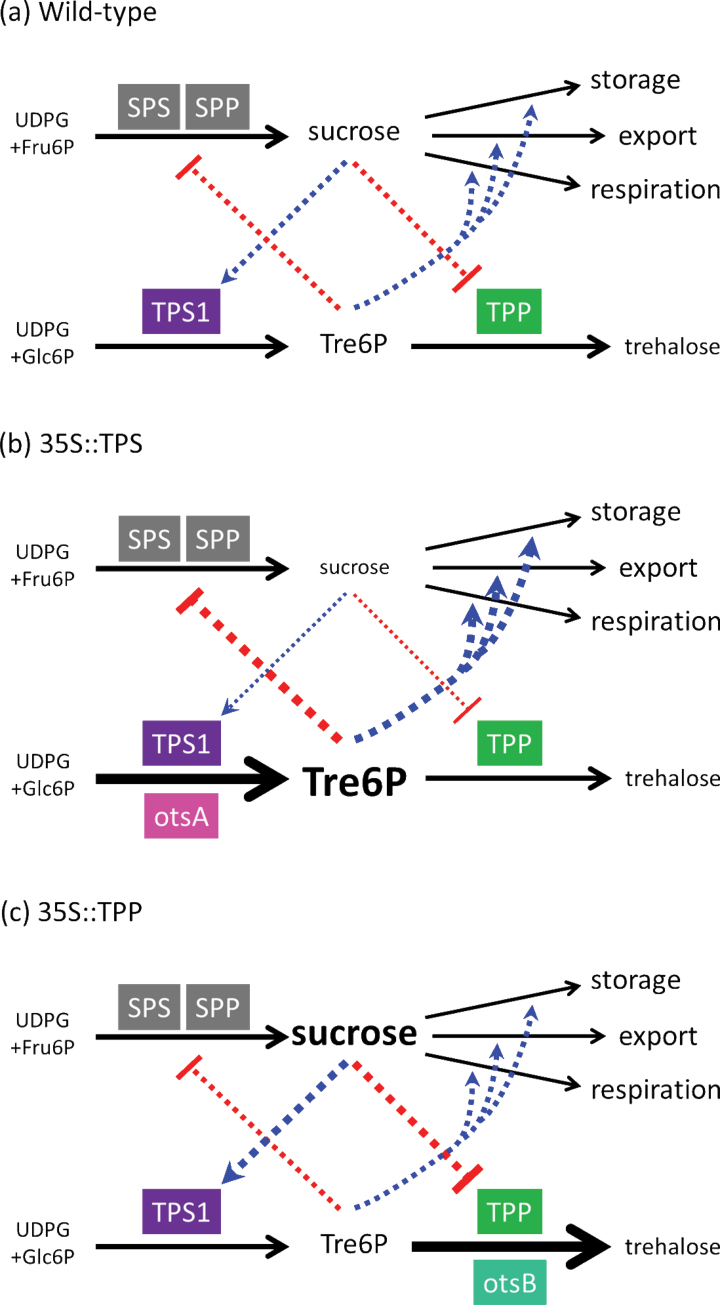
The Tre6P–sucrose nexus in WT, 35S::TPS, and 35S::TPP plants. Blue and red dashed lines show putative activation and inhibition, respectively. Bidirectional control of Tre6P levels by sucrose and vice versa maintains a constant Tre6P:sucrose ratio. In 35::TPS plants, unregulated TPS (otsA) activity increases Tre6P, pushing down sucrose levels, resulting in a high Tre6P:sucrose ratio. In 35S::TPP plants, elevated sucrose compensates for the constant depletion of Tre6P by the unregulated TPP (otsB), resulting in near-WT levels of Tre6P but a low Tre6P:sucrose ratio.

The maintenance of a strong correlation between Tre6P and sucrose in the independent 35S::TPS and 35S::TPP lines implies that Tre6P changes in response to fluctuations in sucrose levels and vice versa, i.e. there is bidirectional regulation of Tre6P and sucrose (Fig. 8). In the 35S::TPS plants, the overexpressed TPS drives higher rates of Tre6P synthesis and an increase in Tre6P levels. According to the bidirectional model, high Tre6P would trigger responses in the plant to lower the level of sucrose and subsequently Tre6P. Sucrose is indeed lower in these plants, but the endogenous mechanism that would normally pull Tre6P down in response to lower sucrose levels is overridden by the constitutively active bacterial TPS. As a result, the plant maintains a relatively constant Tre6P:sucrose ratio, because the mechanism linking sucrose back to Tre6P is still operating, but the ratio is set to a higher value with sucrose levels lower than in WT plants. The 35S::TPP plants appear to compensate for the constant removal of Tre6P by the overexpressed TPP by operating with elevated levels of sucrose, which drive the endogenous regulatory mechanisms to increase Tre6P levels. These opposing forces reach a balance in which the level of Tre6P is close to WT plants, but to maintain this level the plants have a higher sucrose content and lower Tre6P:sucrose ratio.

In conclusion, we propose that the ratio of Tre6P:sucrose is a critical parameter for the plant, and forms part of a homeostatic mechanism to maintain sucrose levels within a range that is appropriate for the cell type and stage of development. Constitutive overexpression of TPS or TPP perturbs, but does not break, the Tre6P–sucrose nexus. However, an enforced change in the Tre6P:sucrose ratio to an inappropriate level has profound consequences for the growth and development of the plant.

## Supplementary data

Supplementary Fig. S1. Correlation of Tre6P with sucrose and other metabolites in *A. thaliana* seedlings exogenously supplied with sucrose.

Supplementary Fig. S2. Correlation of Tre6P with sucrose and other metabolites in *A. thaliana* seedlings exogenously supplied with sucrose or hexose sugars.

Supplementary Fig. S3. Hexose phosphate and UDPG content of *A. thaliana* seedlings exogenously supplied with sucrose and 2-deoxyglucose.

Supplementary Fig. S4. Correlation of Tre6P with sucrose, glucose and fructose in *A. thaliana* seedlings exogenously supplied with disaccharide sugars, glucose, and glucose analogues.

Supplementary Fig. S5. Correlation of Tre6P with other metabolites in *A. thaliana* seedlings treated with mannoheptulose.

Supplementary Fig. S6. Correlation of Tre6P with sugars in nitrogen- and sulphate- starved *A. thaliana* seedlings resupplied with the missing nutrient.

Supplementary Fig. S7. Morphology of wild-type, 35S::TPS, and 35S::TPP *A. thaliana* plants grown in different photoperiods.

Supplementary Fig. S8. Relative growth rates and leaf morphological traits of wild-type, 35S::TPS and 35S::TPP plants grown in different photoperiods.

Supplementary Fig. S9. Metabolite content of wild-type, 35S::TPS, and 35S::TPP plants grown in different photoperiods.

Supplementary Fig. S10. Effect of cordycepin on sucrose-induced changes in *TPS*, *TPP* and *TREHALASE* transcripts in *A. thaliana* seedlings.

Supplementary Fig. S11. Effect of sucrose and cycloheximide on TPS1 protein abundance and Tre6P content of *A. thaliana* seedlings.

Supplementary Fig. S12. Effect of protein kinase and protein phosphatase inhibitors on sucrose-induced changes in the Tre6P content of *A. thaliana* seedlings.

Supplementary Fig. S13. Correlation of Tre6P with other metabolites in *A. thaliana* seedlings treated with MG132.

Supplementary Table S1. Effect of nutrient resupply on Tre6P and sucrose content of N-, P-, and S-starved *A. thaliana* seedlings.

Supplementary Table S1. Effect of nutrient resupply on Tre6P and sucrose content of N-, P-, and S-starved *A. thaliana* seedlings.

Supplementary Table S2. Effect of α-amanitin and cordycepin on transcript levels of sucrose-inducible genes.

Supplementary Table S3. Effect of sucrose resupply on ribosomal occupancy of *TPS*, *TPP*, and *TRE* transcripts in C-starved *A. thaliana* seedlings.

Supplementary Table S4. Analysis of commercially supplied T6P.

Supplementary Table S5. Primers used for real-time qRT-PCR analysis.

Supplementary Methods S1. Mass spectrometric analysis of Tre6P standards

Supplementary Methods S2. Real-time qRT-PCR analysis and polysome loading analysis

Supplementary Data

## Abbreviations:

2-DOG: 2-deoxyglucose
3-OMG: 3-*O*-methylglucose
ED: end of the day
EN: end of the night
FN: full nutrition
Fru6P: fructose 6- phosphate
Glc6P: glucose 6-phosphate
LC-MS/MS: liquid chromatography coupled to tandem mass spectrometry
qRT-PCR: quantitative reverse transcription-PCR
SD: standard deviation
TPP: trehalose-phosphate phosphatase
TPS: trehalose-phosphate synthase
Tre6P: trehalose 6-phosphate
UDPG: uridine 5’-diphosphoglucose.
