# Supplementary material for: The sucrose–trehalose 6-phosphate (Tre6P) nexus: specificity and mechanisms of sucrose signalling by Tre6P
Source: J Exp Bot. 2014 Jan 13;65(4):1051–68. doi: 10.1093/jxb/ert457 (PMC3935566; doi:10.1093/jxb/ert457)
Supplement: Supplementary Data [file supp_65_4_1051__index.html]

The sucrose–trehalose 6-phosphate (Tre6P) nexus: specificity and mechanisms of sucrose signalling by Tre6P — The sucrose–trehalose 6-phosphate (Tre6P) nexus: specificity and mechanisms of sucrose signalling by Tre6P — Supplementary Data 

# The sucrose–trehalose 6-phosphate (Tre6P) nexus: specificity and mechanisms of sucrose signalling by Tre6P

## Supplementary Data

Data files

**Files in this Data Supplement:**

- Supplementary Data - Supplementary Data
