# Supplementary material for: The sucrose–trehalose 6-phosphate (Tre6P) nexus: specificity and mechanisms of sucrose signalling by Tre6P
Source: J Exp Bot. 2014 Jan 13;65(4):1051–68. doi: 10.1093/jxb/ert457 (PMC3935566; doi:10.1093/jxb/ert457)
Supplement: Supplementary Data [file supp_ert457_jexbot113712_file001.pdf]

SUPPORTING EXPERIMENTAL PROCEDURES

Methods S1. Mass spectrometric analysis of Tre6P standards

The early commercial supplies of Tre6P from Sigma-Aldrich (available up to 2010) were found to contain only 65-82% Tre6P (Lunn *et al.*, 2006). Fourier-transform mass spectrometric analysis (Giavalisco *et al.*, 2008) revealed that the most abundant contaminants were C_{16} and C_{18} fatty acids and hexose-phosphates, with smaller amounts of pentose-phosphates, other sugar-phosphates, short- and long-chain fatty acids, and artificial tissue-solubilizing agent and several other detergent-like molecules (Supporting Table S4). In a newer batch of Tre6P (supplied by Sigma-Aldrich in 2011) analysed by UPLC-MS (Giavalisco *et al.*, 2011), the monovalent deprotonated form of Tre6P $[\text{Tre6P-H}^{+}]^{-}$ was by far the dominant species, accompanied by a few minor peaks, all of which could be assigned to ionic forms of Tre6P in various states of hydration or association with potassium ions (data not shown). Spectrophotometric assay using phosphotrehalase (Lunn *et al.*, 2006) confirmed that the newer batches of Tre6P that we used for calibration of the LC-MS/MS assay are close to 100% purity.

Methods S2. Real Time RT-qPCR and polysome loading analysis

RNA was extracted using either Trizol reagent (www.invitrogen.com) or an RNeasy Mini Prep Kit (www.qiagen.com). Following quality control checks and removal of genomic DNA by DNase treatment (Czechowski *et al.*, 2005), RNA was reverse transcribed from an oligo(dT)18 primer using SuperScript™ III reverse transcriptase (www.invitrogen.com). Real-Time amplification was performed in optical 384-well plates using Power SYBR® Green reagent and an ABI PRISM® 7900 HT Sequence Detection System (www.appliedbiosystems.com) (Czechowski *et al.*, 2005). Primers are listed in Supporting Table S5. The PCR efficiencies of primers were estimated using the LinRegPCR software (Ramakers *et al.*, 2003). Threshold cycle (C_T) value were normalised using *GAPDH* as the internal reference gene and are presented as fold-change relative to carbon-starved seedling samples.

Polysomes were extracted and separated from non-polysomal ribosomes on sucrose density gradients as described by Piques *et al.* (2009). Polysomal and non-polysomal fractions were pooled separately. RNA was extracted from each fraction and spiked with *in vitro* transcribed polyadenylated mRNAs from *Bacillus subtilis* genes (Poly-A RNA Control

Kit; www.affymetrix.com) for normalization (Piques *et al*., 2009), before measurement of mRNA abundance by Real-Time RT-qPCR as described above.

Methods S3. Leaf morphological analysis

Fully expanded leaves were harvested, weighed (fresh weight), photographed alongside a ruler, dried at 70°C and weighed again (dry weight) to determine leaf dry matter content (LDMC). Leaf area was determined from the photographs using ImageJ and used to calculate specific leaf area (SLA). Relative growth rate was calculated from the fresh weight. Leaf thickness was claculated from LDMC and SLA as described by Vile *et al*. (2005)

References

Czechowski, T., Stitt, M., Altmann, T., Udvardi, M.K. and Scheible, W.R. (2005) Genome-wide identification and testing of superior reference genes for transcript normalization in Arabidopsis. *Plant Physiol.* **139**, 5–17.

Giavalisco, P., Hummel, J., Lisec, J., Inostroza, A.C., Catchpole, G. and Willmitzer, L. (2008) High-resolution direct infusion-based mass spectrometry in combination with whole ^{13}C metabolome isotope labeling allows unambiguous assignment of chemical sum formulas. *Anal. Chem.* **80**, 9417–9425.

Giavalisco P, Li Y, Matthes A, Eckhardt A, Hubberten HM, Hesse H, Segu S, Hummel J, Köhl K, Willmitzer L. (2011) Elemental formula annotation of polar and lipophilic metabolites using ^{13}C, ^{15}N and ^{34}S isotope labelling, in combination with high-resolution mass spectrometry. *Plant J.* **68**, 364–76.

Lunn, J.E., Feil, R., Hendriks, J.H., Gibon, Y., Morcuende, R., Osuna, D., Scheible, W.R., Carillo, P., Hajirezaei, M.R. and Stitt, M. (2006). Sugar-induced increases in trehalose 6-phosphate are correlated with redox activation of ADPglucose pyrophosphorylase and higher rates of starch synthesis in *Arabidopsis thaliana*. *Biochem. J.* **397**, 139–148.

Piques, M., Schulze, W.X., Höhne, M., Usadel, B., Gibon, Y., Rohwer, J. and Stitt, M. (2009) Ribosome and transcript copy numbers, polysome occupancy and enzyme dynamics in Arabidopsis. Mol. Syst. Biol. **5**, 314.

Ramakers, C., Ruijter, J.M., Deprez, R.H. and Moorman, A.F. (2003) Assumption-free analysis of quantitative real-time polymerase chain reaction (PCR) data. *Neurosci. Lett.* **339**, 62–66.

Vile, D., Garnier, E., Shipley, B., Laurent, G., Navas, M.L., Roumet, C., Lavorel, S., Díaz, S., Hodgson, J.G., Lloret, F., Midgley, G.F., Poorter, H., Rutherford, M.C., Wilson, P.J. and Wright, I.J. (2005) Specific leaf area and dry matter content estimate thickness in laminar leaves. *Ann. Bot.* **96**, 1129–1136.

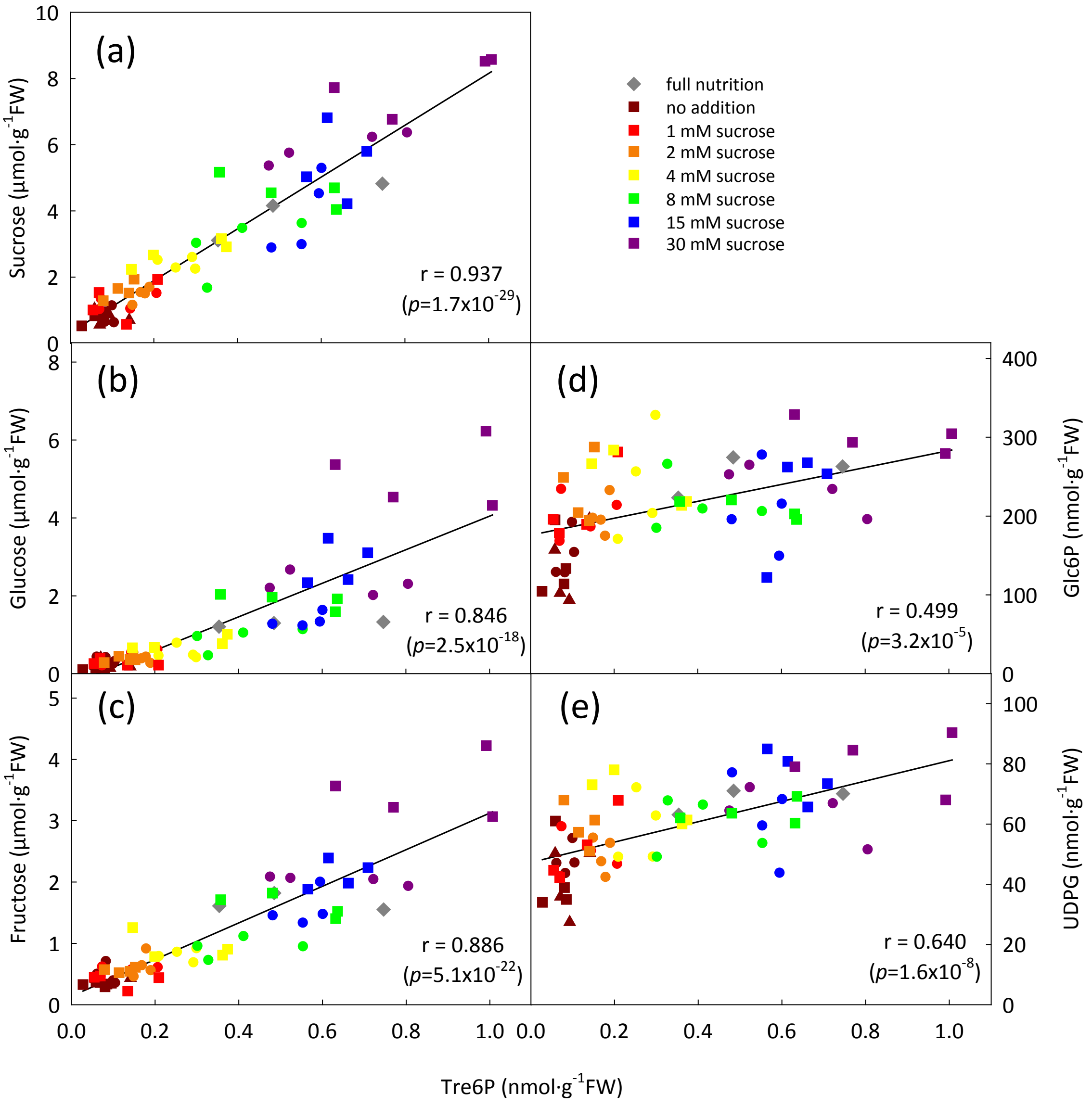

Supporting Figure S1. Correlation of Tre6P with sucrose and other metabolites in *Arabidopsis thaliana* seedlings exogenously supplied with sucrose.

Sucrose was supplied to C-starved 9-day-old seedlings at final concentrations of 1-30 mM. Samples were harvested before (▲), and at 75 min. (●) or 180 min. (■) after sucrose addition. Non-starved control seedlings were grown in full nutrition medium containing 15 mM sucrose (♦). The Tre6P content of individual samples is plotted against: (a) sucrose, (b) glucose, (c) fructose, (d) glucose 6-phosphate and (e) UDP-glucose. The Pearson correlation coefficient (r) for each metabolite pair is shown (*p*-values in parentheses). Data are from the same experiment shown in Fig. 1.

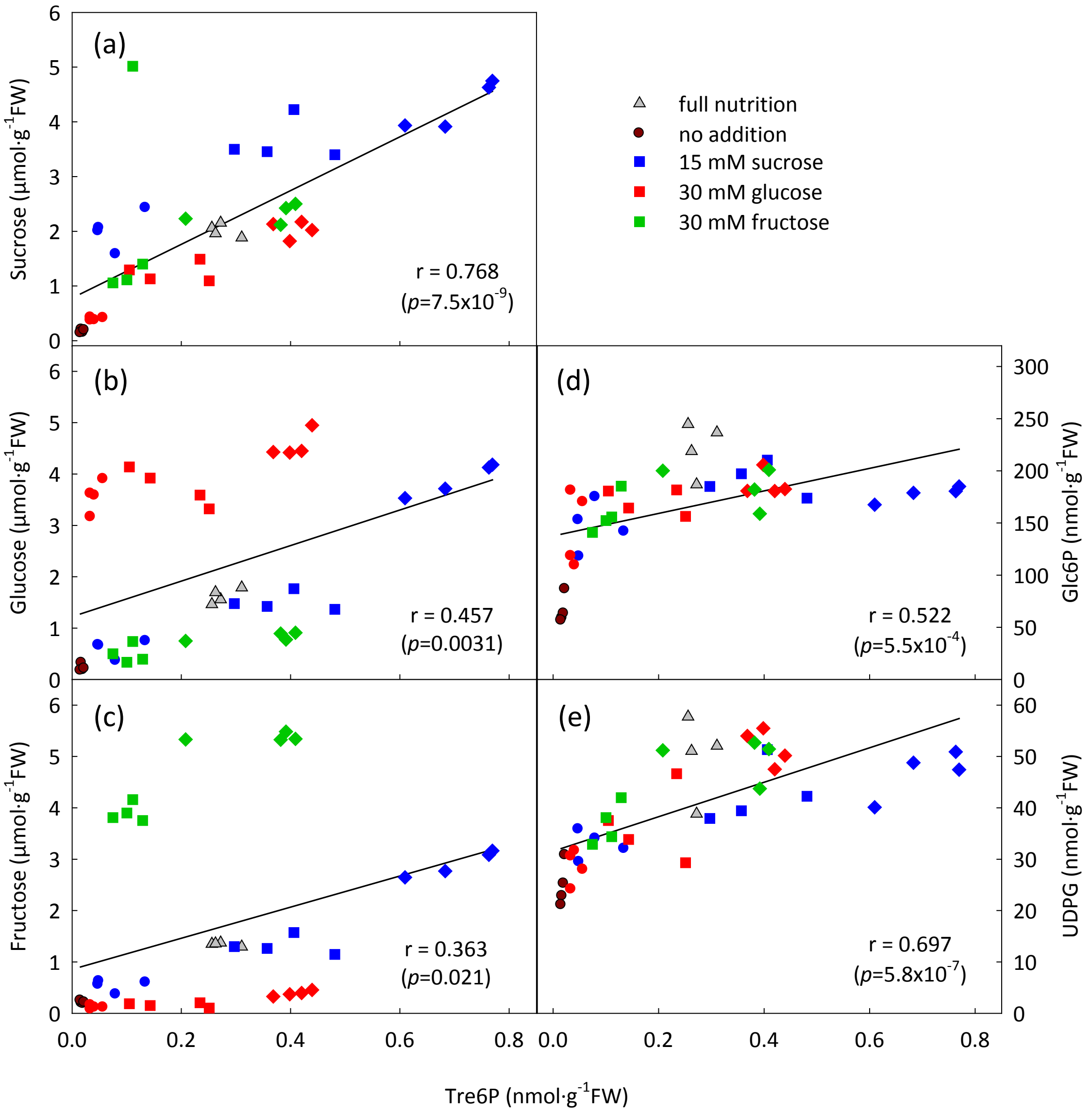

Supporting Figure S2. Correlation of Tre6P with sucrose and other metabolites in *Arabidopsis thaliana* seedlings exogenously supplied with sucrose or hexose sugars.

Sucrose (15 mM), glucose (30 mM) or (fructose (30 mM) were supplied to C-starved 9-d-old seedlings at the final concentrations indicated in parentheses. Samples were harvested before (open circles), and at 30 (closed circles; not fructose), 75 (squares) or 180 min. (diamonds) after sugar addition. Non-starved control samples (triangles) were grown in full nutrition medium containing 15 mM sucrose. The Tre6P content of individual samples is plotted against: (a) sucrose, (b) glucose, (c) fructose, (d) glucose 6-phosphate and (e) UDP-glucose. The Pearson correlation coefficient (r) for each metabolite pair is shown (*p*-values in parentheses). Data are from the same experiment shown in Fig. 2.

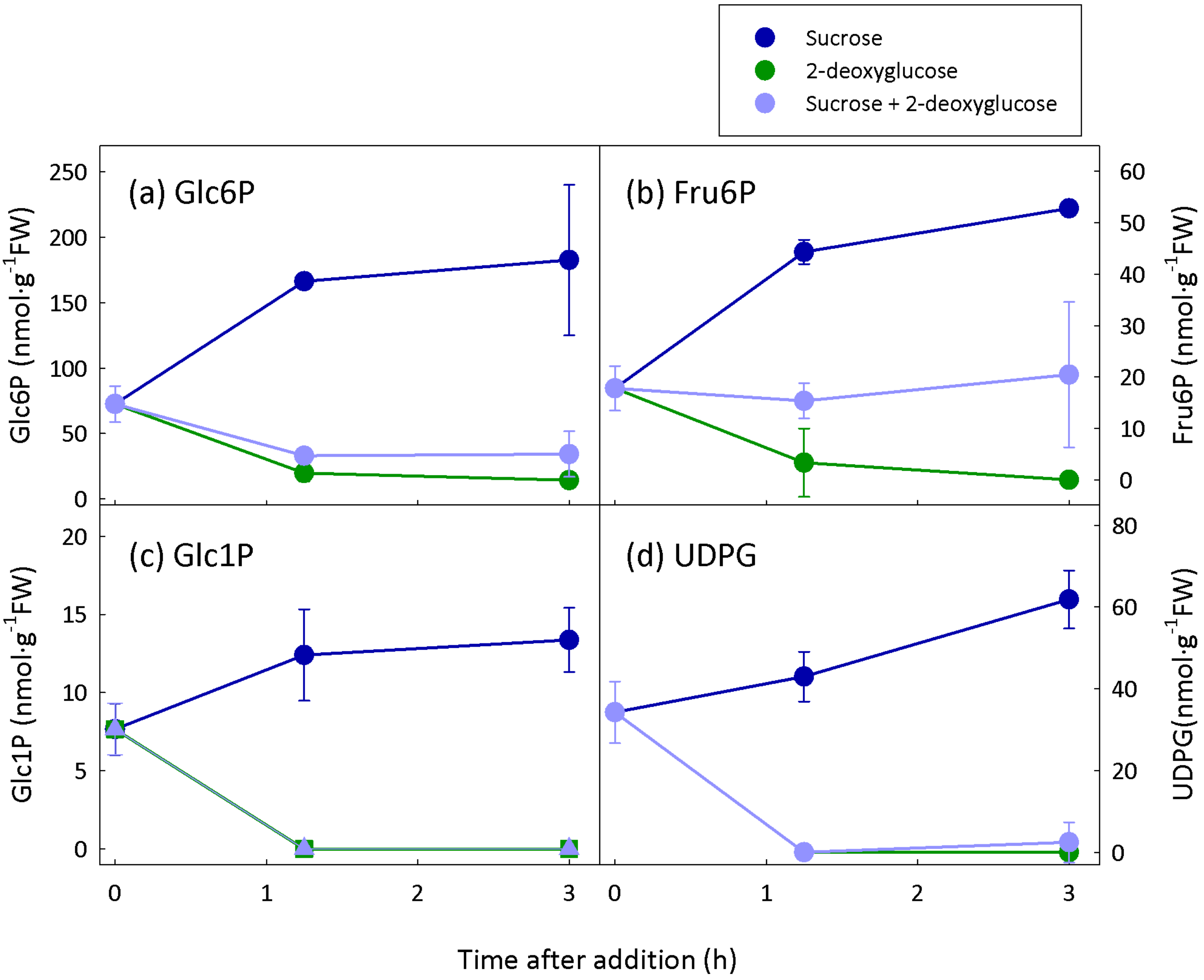

Supporting Figure S3. Hexose-phosphate and UDPG content of *Arabidopsis thaliana* seedlings exogenously supplied with sucrose and 2-deoxyglucose.

Sucrose (± 2-deoxyglucose) or 2-deoxyglucose alone were supplied to C-starved 9-d-old seedlings at final concentrations of 15 mM. Samples were harvested before sugar addition and 75 or 180 min. after sugar addition for metabolite measurments. Values are mean ± S.D. (n = 4). Samples were from the same experiment shown in Fig. 3.

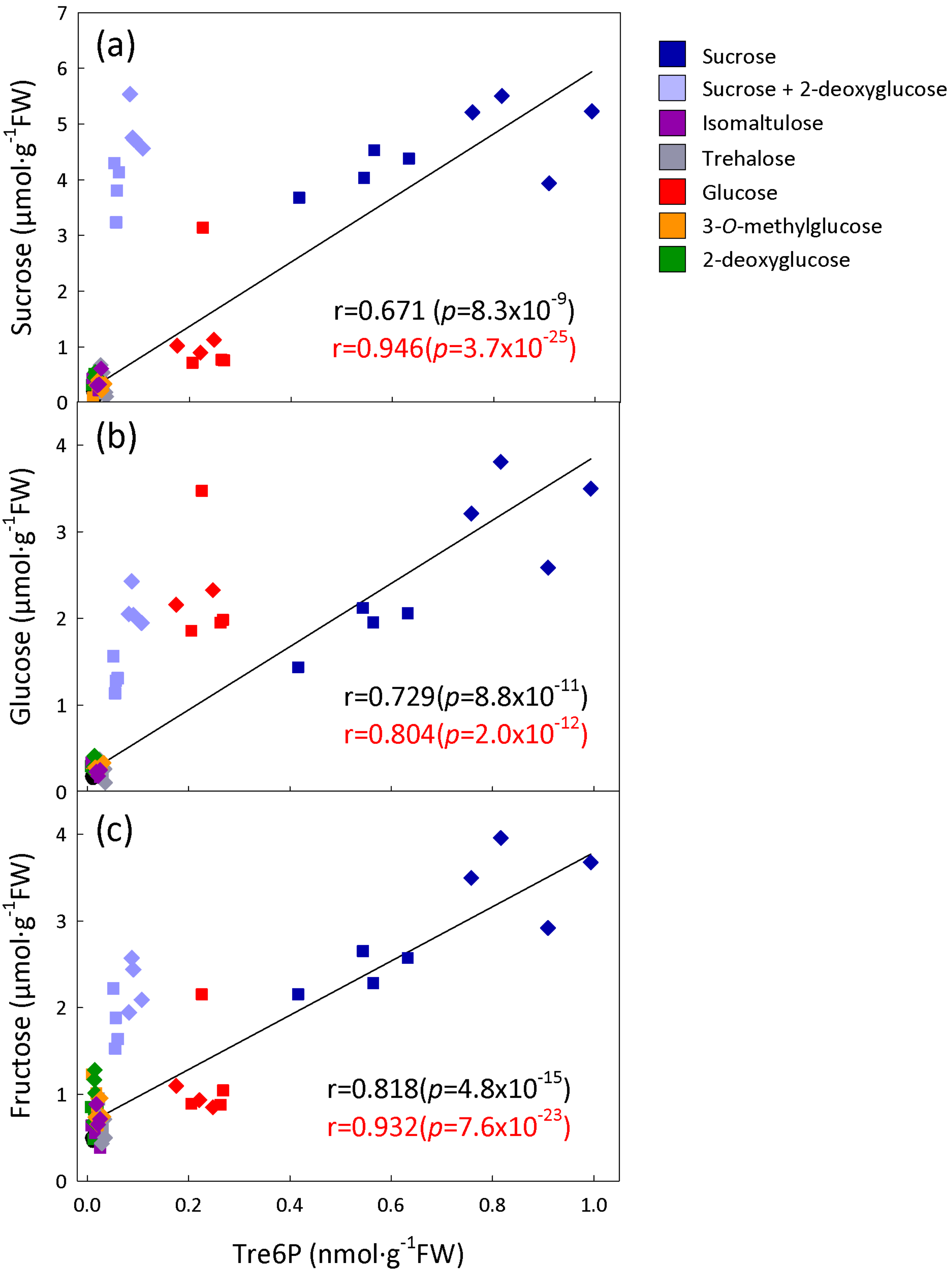

Supporting Figure S4. Correlation of Tre6P with sucrose, glucose and fructose in *Arabidopsis thaliana* seedlings exogenously supplied with disaccharide sugars, glucose and glucose analogues.

Sucrose (± 2-deoxyglucose), isomaltulose, trehalose, glucose, 3-*O*-methylglucose or 2-deoxyglucose (all at final concentrations of 15 mM) were supplied to C-starved 9-d-old seedlings. Samples were harvested before (circles), and at 75 min. (squares) or 180 min. (diamonds) after sugar addition. The Tre6P content of individual samples is plotted against: (a) sucrose, (b) glucose, and (c) fructose. The Pearson correlation coefficient (r) for each metabolite pair is shown with (black) or without (red) the samples supplied with sucrose + 2-deoxyglucose (*p*-values in parentheses). Lines show linear regression without the latter samples. Data are from the same experiment shown in Fig. 3.

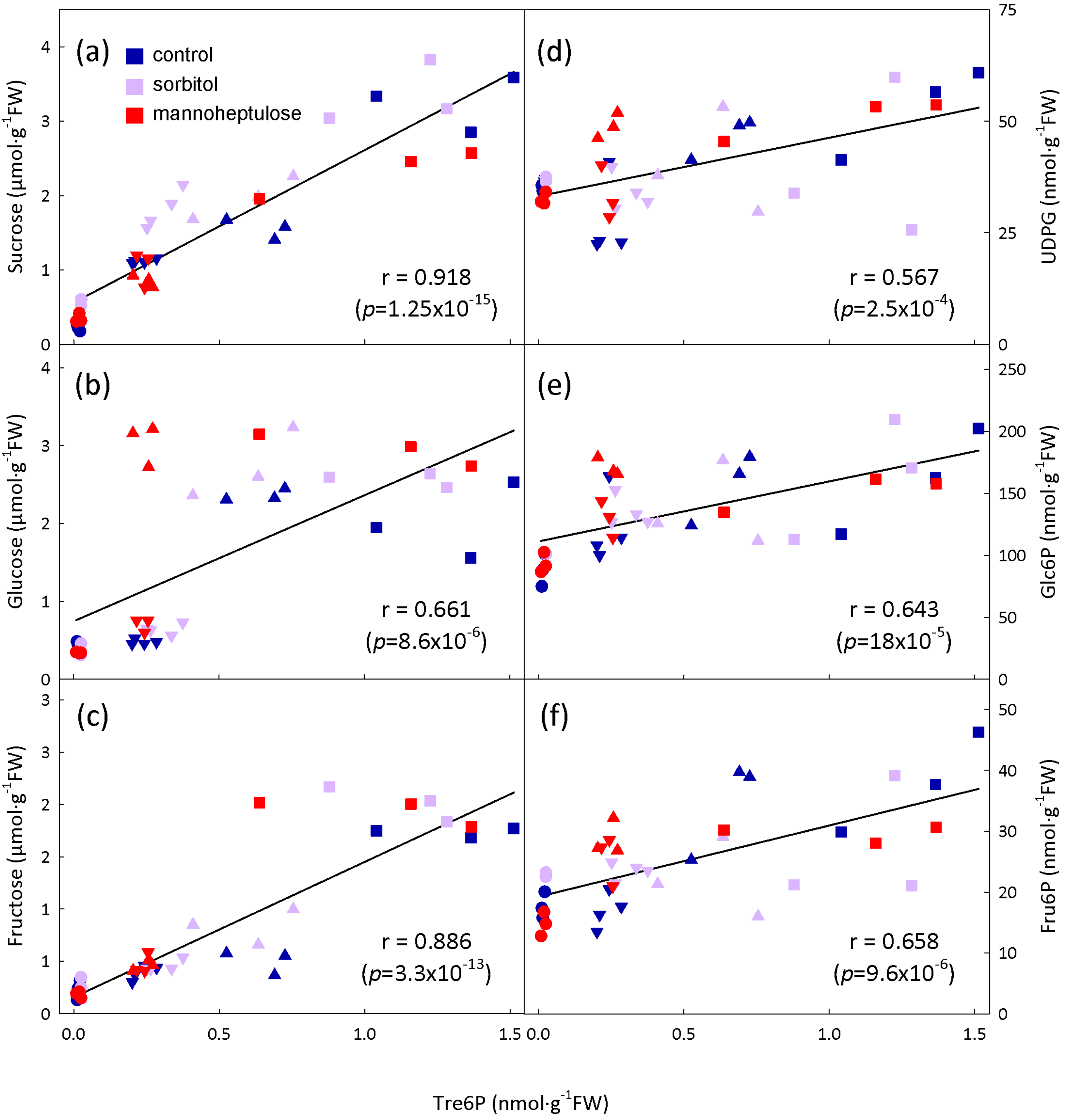

Supporting Figure S5. Correlation of Tre6P with other metabolites in *Arabidopsis thaliana* seedlings treated with mannoheptulose.
C-starved 9-d-old seedlings were incubated with 100 mM sorbitol, 100 mM mannoheptulose or with no addition (control) for 1 h before supplying 7.5 mM sucrose (■), 15 mM glucose (▲) or 15 mM galactose (▼). Samples were harvested before (●) and 3 h after sugar addition. The Tre6P content of individual samples is plotted against: (a) sucrose, (b) glucose, (c) fructose, (d) UDP-glucose, (e) Glc6P, and (f) Fru6P. Lines show linear regression of all samples. The Pearson correlation coefficients (r) is shown for each metabolite pair (*p*-values in parentheses). Data are from the same experiment shown in Fig.4.

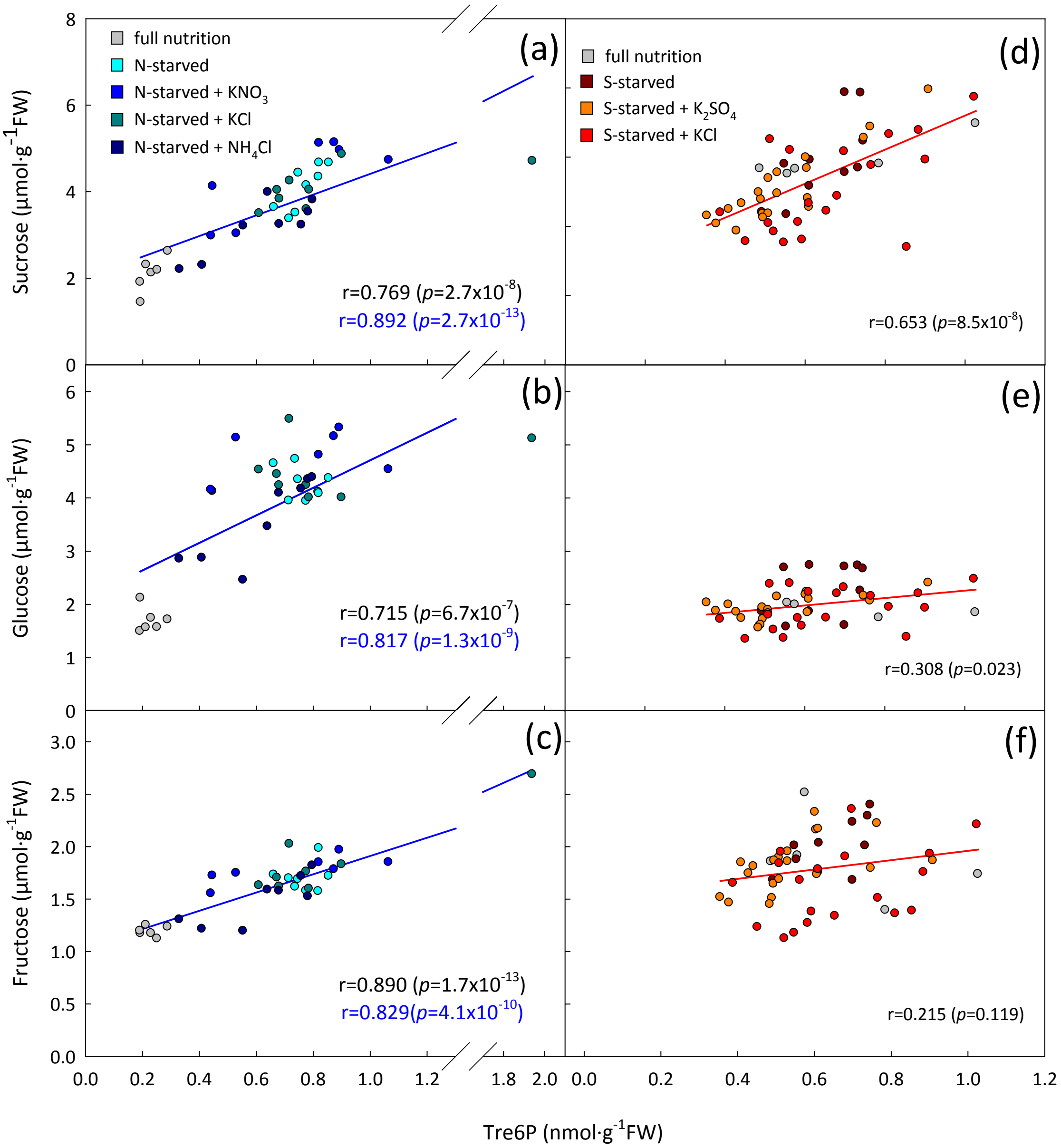

Supporting Figure S6. Correlation of Tre6P with sugars in nitrogen- and sulphate- starved *Arabidopsis thaliana* seedlings resupplied with the missing nutrient.

Seedlings were grown in axenic culture with full nutrition medium (Scheible *et al.*, 2004) for 7 days and then transferred to N- or S-starvation medium. After two days, the missing nutrient (KNO_3, NH_4Cl or K_2SO_4) or KCl (control) was resupplied to the seedlings, and samples were harvested at 30 min or 3 h after the addition for metabolite measurements. The Tre6P content of individual samples is plotted against: (a,d) sucrose, (b,e) glucose, and (c,f) fructose. Lines show linear regression of all samples. The Pearson correlation coefficient (r) for each metabolite pair is shown (*p*-values in parentheses). In a-c, r values calculated with the N-starved(+KCl) outlier (1.94 nmol g^{-1}FW Tre6P) excluded are shown in blue). Data are from the same experiment shown in Supporting Table S1.

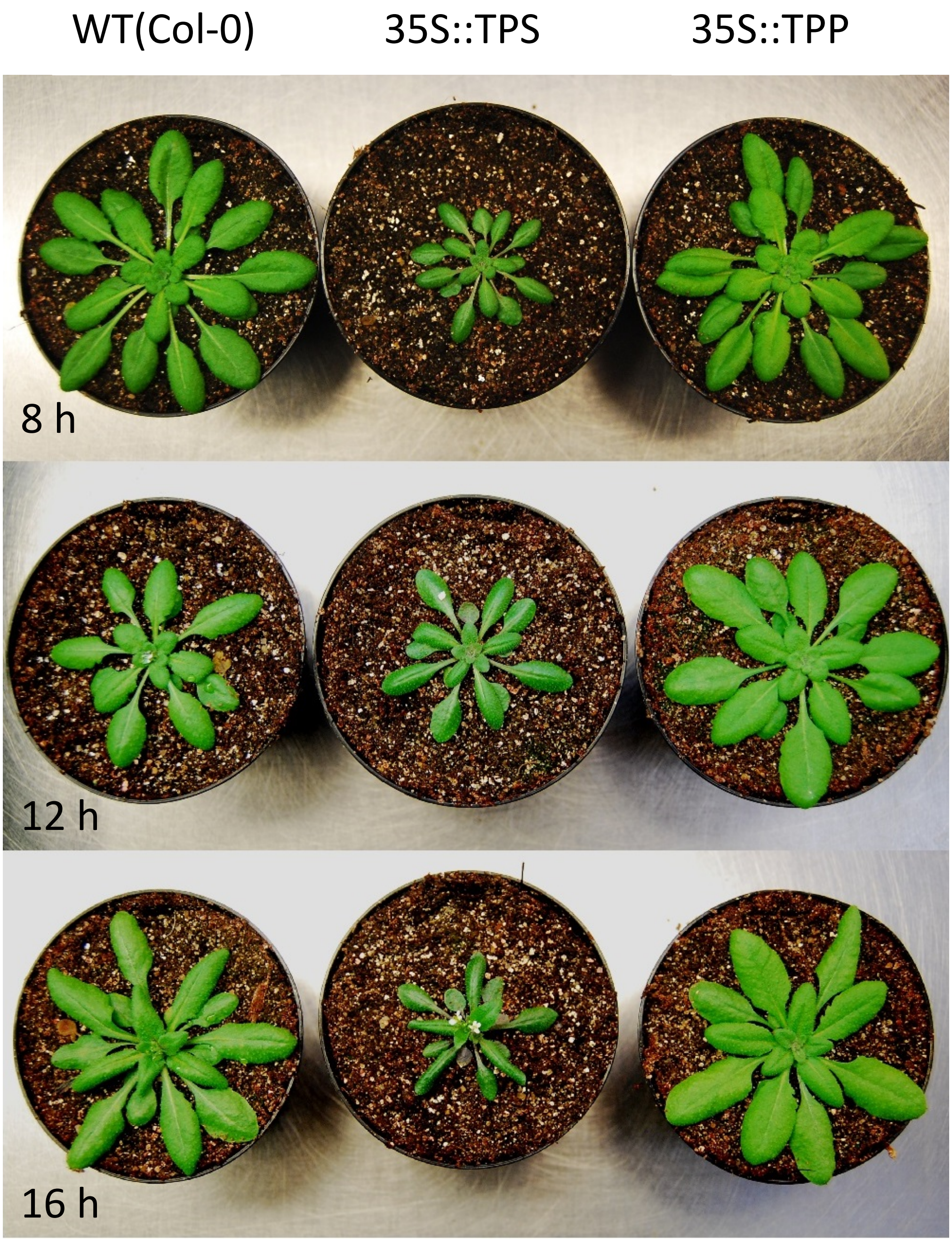

Supporting Figure S7. Morphology of wild-type, 35S::TPS and 35S::TPP *Arabidopsis thaliana* plants grown in different photoperiods. Plants were grown under 8, 12 and 16-h photoperiods with an irradiance of 160 µE m^{-2} s^{-1} and constant temperature of 20°C. The plants shown were 46 d old (8-h photoperiod) or 35 d old (12 and 16-h photoperiods).

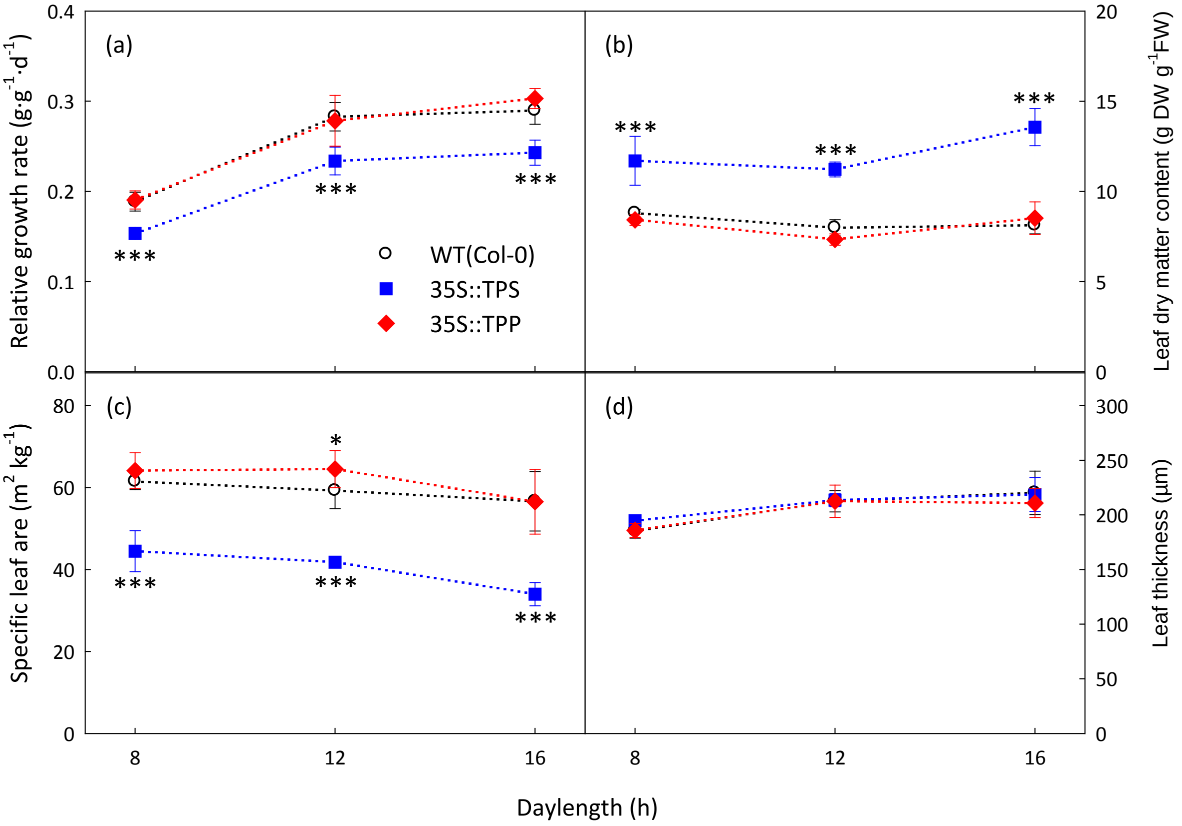

Supporting Figure S8. Relative growth rates and leaf morphological traits of wild-type, 35S::TPS and 35S::TPP plants grown in different photoperiods. Plants were grown under 8, 12 and 16-h photoperiods with an irradiance of 160 µE m^{-2} s^{-1} and constant temperature of 20°C. Rosettes from 46-d-old plants (8-h photoperiod) or 35-d-old plants (12 and 16-h photoperiods) were harvested for measurement of (a) relative growth rate, (b) leaf dry matter content (LDMC) and (c) specific leaf area (SLA). LDMC and SLA were used to calculate (d) leaf thickness as described by Vile *et al*. (2005). Values are mean ± SD (n=8). Asterisks indicate significant differences (Student's t-test) from wild-type. *p<0.05, ***p<0.001.

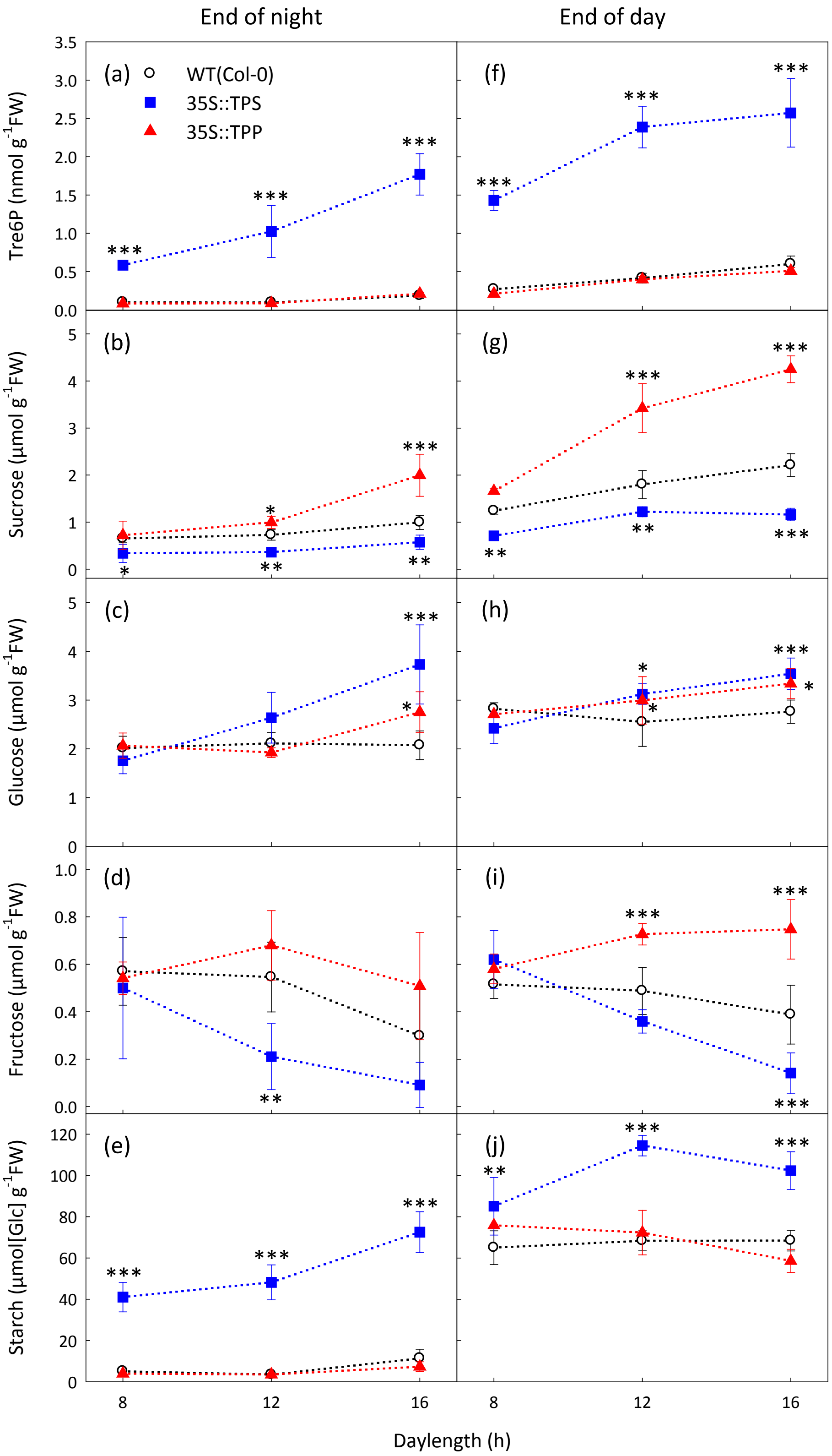

Supporting Figure S9. Metabolite content of wild-type, 35S::TPS and 35S::TPP plants grown in different photoperiods. Rosettes from 25-d-old plants were harvested at the end of the night (a-e) and the end of the day (f-j). Values are mean ± SD (n=5, except 35S::TPP at end of day n=2). Asterisks indicate significant differences (Student's t-test) from wild-type. *p<0.05, **p<0.01, ***p<0.001.

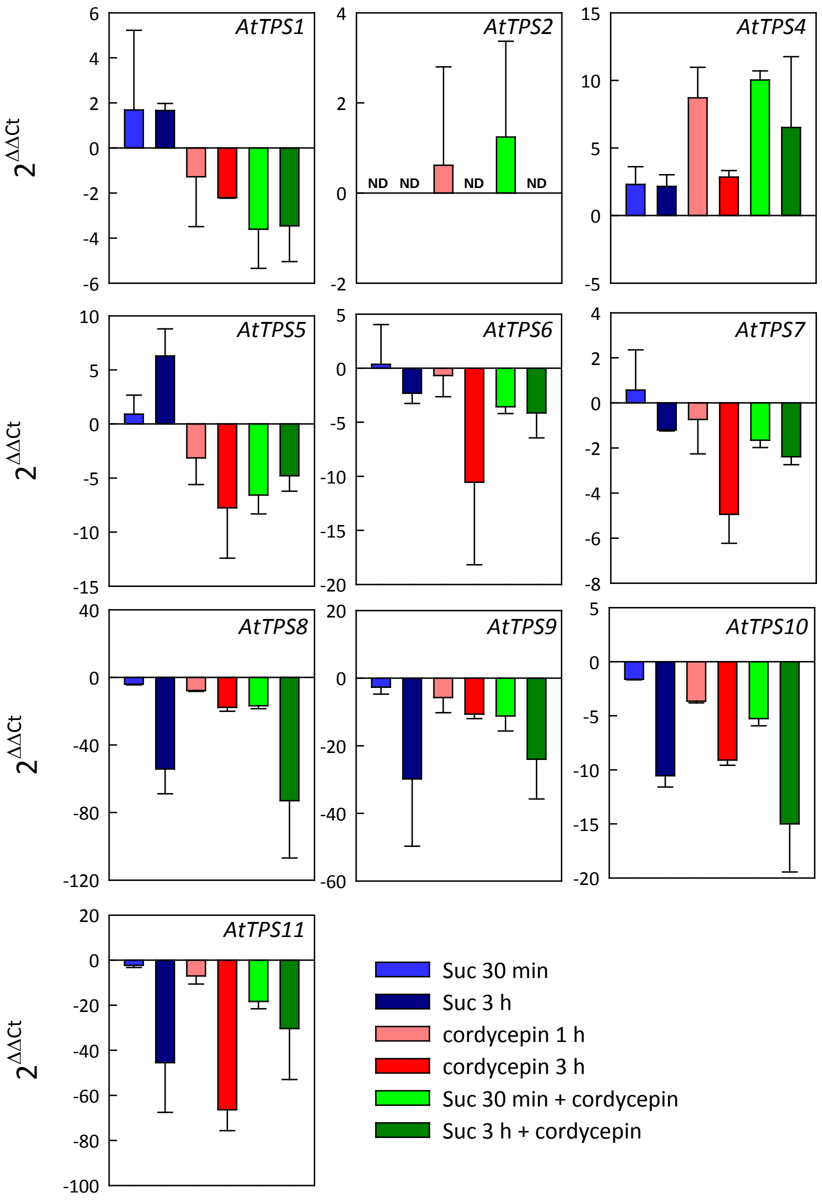

Supporting Figure S10a. Effect of cordycepin on sucrose-induced changes in *TPS*, *TPP* and *TREHALASE* transcripts in *Arabidopsis thaliana* seedlings.

C-starved 9-d-old seedlings were incubated with 0.6 mM cordycepin for 1 h before addition of sucrose (15 mM final concentration). Control samples were not treated with cordycepin. Samples were harvested before and 3 h after sucrose addition for measurement of *TPS*, *TPP* and *TRE* transcript abundance by RT-qPCR. No transcripts were detected for *TPS3*, which is thought to be a pseudogene (Lunn, 2007). Values are mean ± S.D. (n = 4). Samples were from the same experiment shown in Fig. 6. ND, not detected.

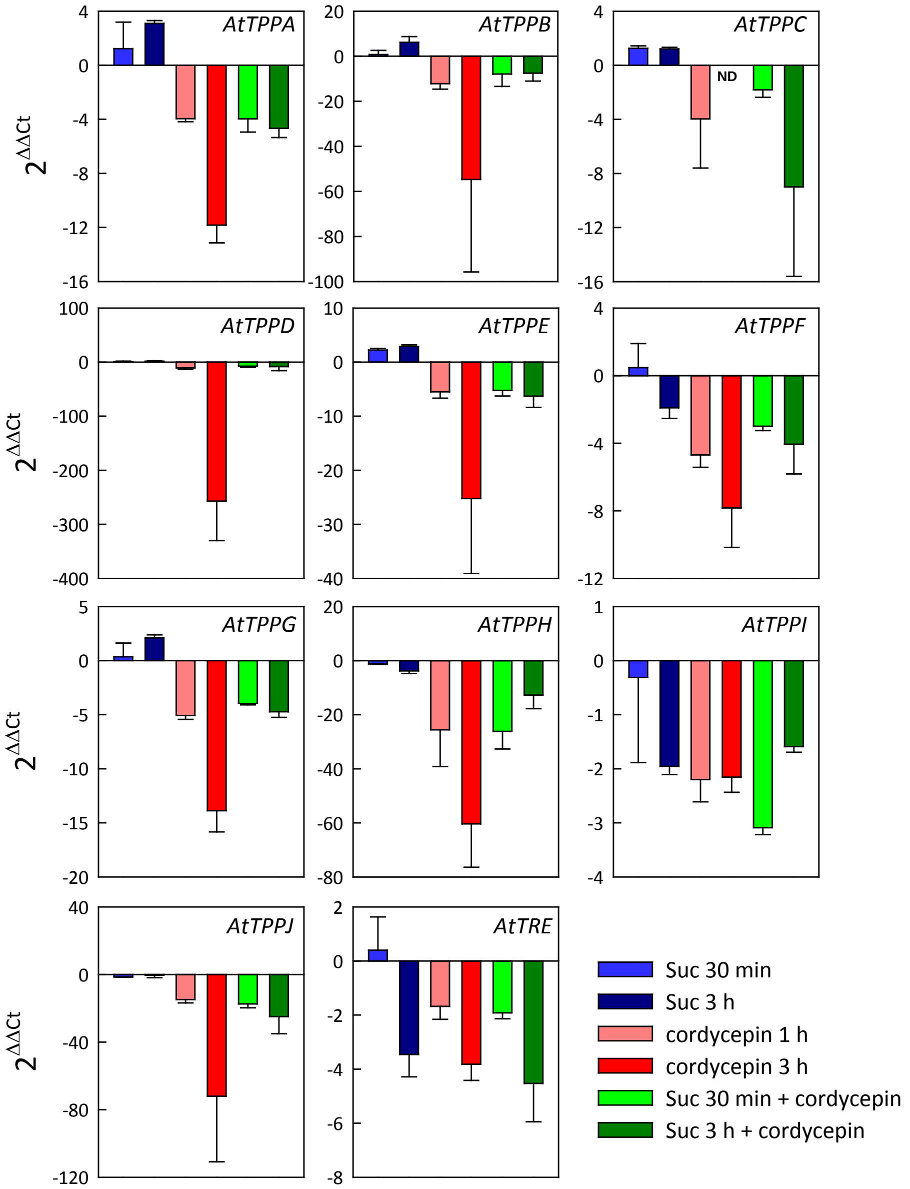

Supporting Figure S10b. Effect of cordycepin on sucrose-induced changes in *TPS*, *TPP* and *TREHALASE* transcripts in *Arabidopsis thaliana* seedlings.

C-starved 9-d-old seedlings were incubated with 0.6 mM cordycepin for 1 h before addition of sucrose (15 mM final concentration). Control samples were not treated with cordycepin. Samples were harvested before and 3 h after sucrose addition for measurement of *TPS*, *TPP* and *TRE* transcript abundance by RT-qPCR. No transcripts were detected for *TPS3*, which is thought to be a pseudogene (Lunn, 2007). Values are mean ± S.D. (n = 4). Samples were from the same experiment shown in Fig. 6. ND, not detected.

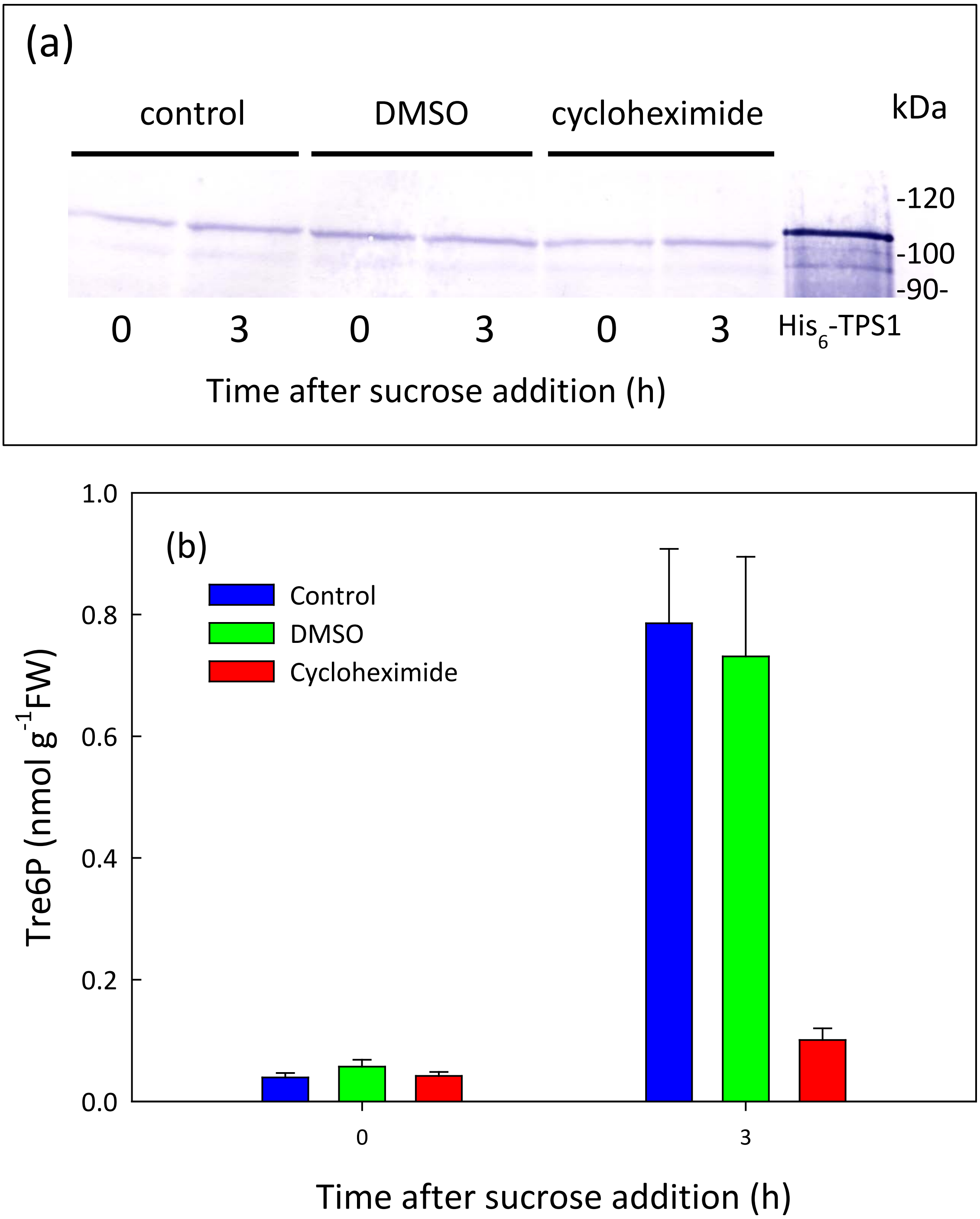

Supporting Figure S11. Effect of sucrose and cycloheximide on TPS1 protein abundance and Tre6P content of *Arabidopsis thaliana* seedlings.

C-starved 9-d-old seedlings were incubated with 100 µM cycloheximide for 1 h before addition of sucrose (15 mM final concentration). Samples were harvested before and 3 h after sucrose addition. Control seedlings were not treated with cycloheximide or were incubated with 4 mM DMSO. (a) Immunoblot of seedling extracts (10 µg protein) probed with α-AtTPS1 antibody. The right hand lane contains 10 ng of His_6-AtTPS1 (109 kDa) heterologously expressed in *E. coli*. (b) Tre6P content measured by LC-MS/MS, values are mean ± S.D. (n = 4).

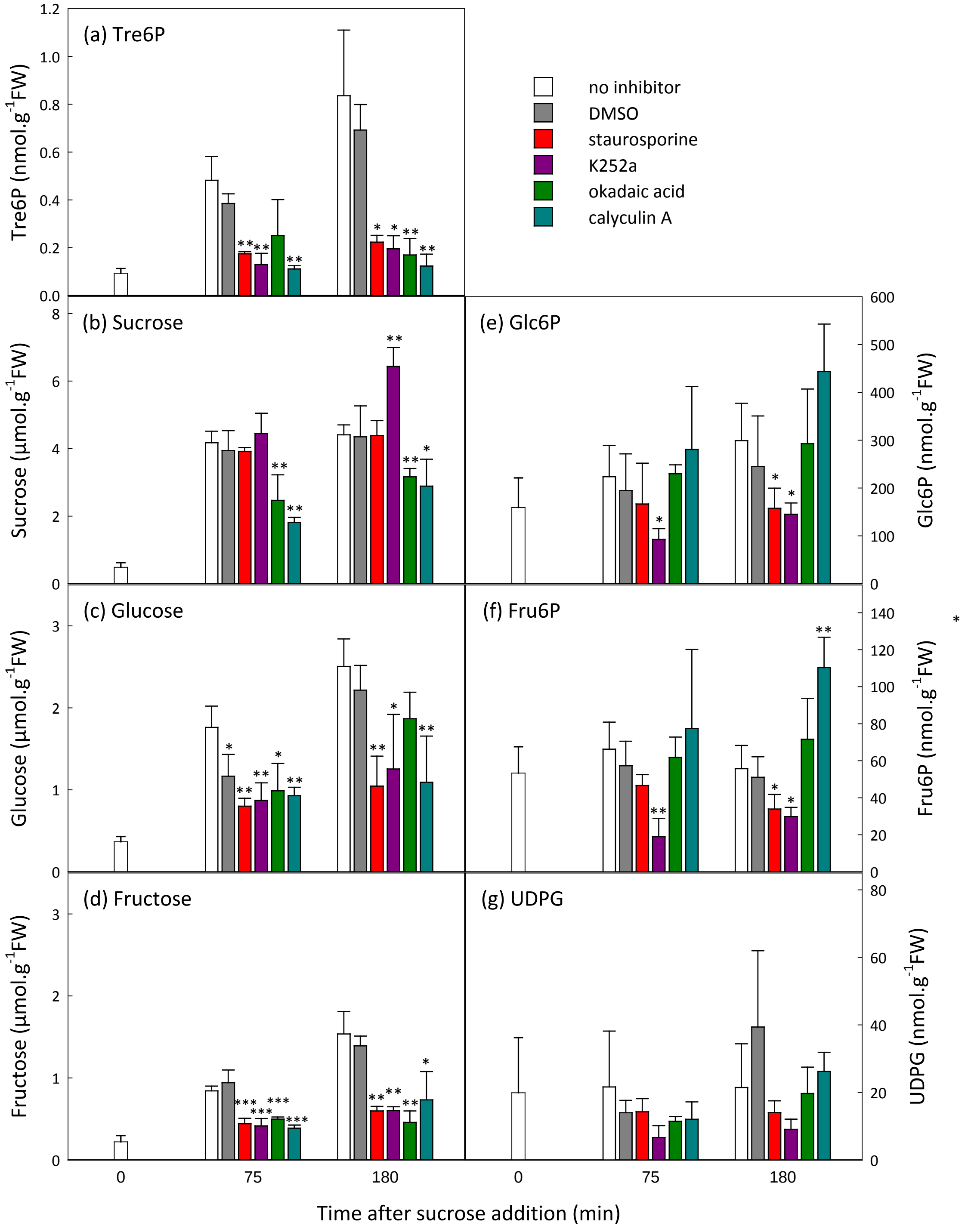

Supporting Figure S12. Effect of protein kinase and protein phosphatase inhibitors on sucrose-induced changes in the Tre6P content of *Arabidopsis thaliana* seedlings.

C-starved 9-d-old seedlings were incubated with 1 µM staurosporine, 1 µM K252a, 167 nM okadaic acid, 55 nM calyculin A for 1 h before supplying sucrose (final concentration 15 mM). Control seedlings were incubated with no inhibitor, or with 14 mM DMSO as solvent control. Samples were harvested before and 75 min or 3 h after sucrose addition for metabolite measurements. Values are mean ± S.D. (n = 4). Significant differences (Student's t-test) between the inhibitor-treated seedlings and the respective controls are indicated by asterisks: *p < 0.05; **p < 0.01.

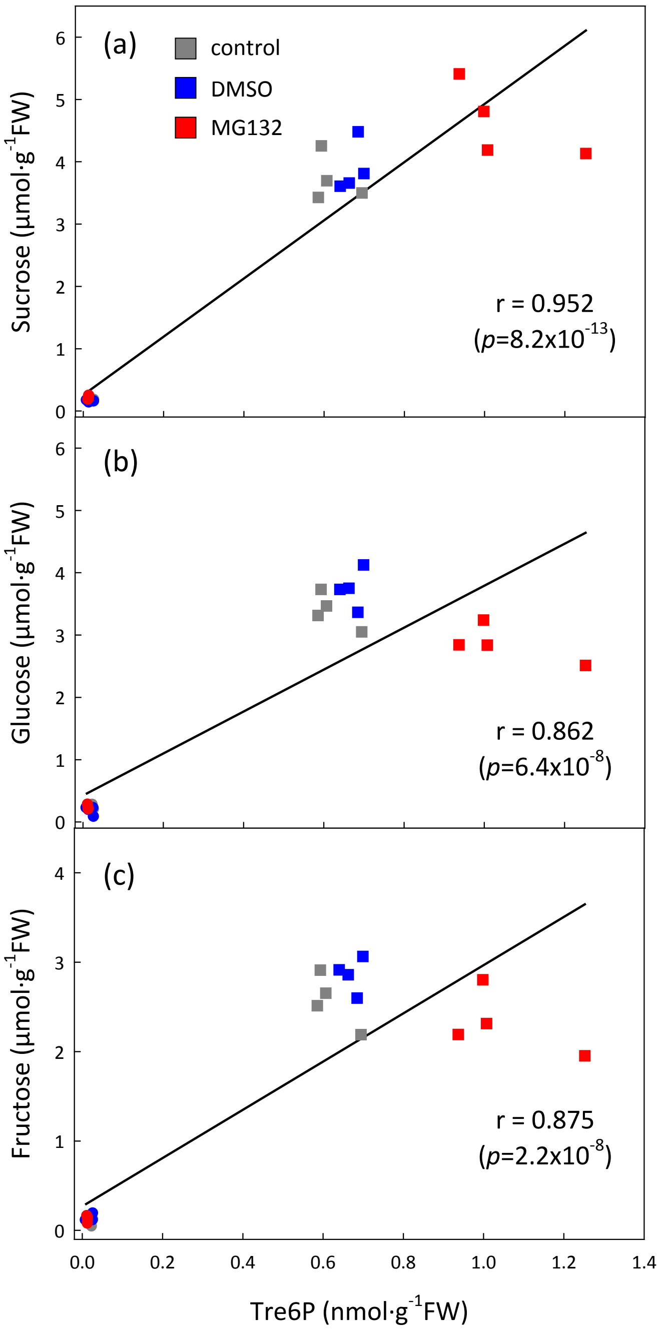

Supporting Figure S13. Correlation of Tre6P with other metabolites in *Arabidopsis thaliana* seedlings treated with MG132.

C-starved 9-d-old seedlings were incubated with 34 mM DMSO or 100 µM MG132 + 34 mM DMSO or with no addition (control) for 1 h before supplying sucrose (15 mM final concentration). Samples were harvested before (●) and 3 h after (■) sucrose addition for metabolite measurements. The Tre6P content of individual samples is plotted against: (a) sucrose, (b) glucose, and (c) fructose. The Pearson correlation coefficient (r) for each metabolite pair is shown (*p*-values in parentheses). Data are from the same experiment shown in Fig. 7.

Supporting Table S1. Effect of nutrient resupply on Tre6P and sucrose content of N, P and S-starved *Arabidopsis thaliana* seedlings. Seedlings were grown in axenic culture with full nutrition medium (Scheible *et al.*, 2004) for 7 days and then transferred to N-, P- or S-starvation medium. After two days, the missing nutrient (KNO_3, NH_4Cl, KH_2PO_4 or K_2SO_4) or KCl (control) was resupplied to the seedlings, and samples were harvested at 30 min or 3 h after the addition for metabolite analysis. Data are mean ± S.D. (n=4 or 5 unless indicated otherwise). Values that are significantly different (Student's *t*-test) from the corresponding N, P or S-starved samples are indicated in bold: $^{a}p<0.05$; $^{b}p<0.01$; $^{c}p<0.001$. n.d., not determined. $^{d}n=2$.

Medium	Addition	Time<br>*min*	Tre6P<br>*nmol·g^{-1}FW*	Sucrose<br>*µmol·g^{-1}FW*
N-starvation				
Full nutrition	none	0	**0.229^{c}± 0.037**	**2.10^{c}± 0.40**
NO_3 starvation	none	0	0.799 ± 0.048	4.39 ± 0.22
NO_3 starvation	none	180	0.734 ± 0.066	3.80 ± 0.60
NO_3 starvation	KCl	30	0.699 ± 0.073	**3.90^{a}± 0.32**
NO_3 starvation	KCl	180	1.073 ± 0.586	4.30 ± 0.60
NO_3 starvation	KNO_3	30	0.920 ± 0.129	4.99 ± 0.23
NO_3 starvation	KNO_3	180	0.578 ± 0.214	3.77 ± 0.95
NO_3 starvation	NH_4Cl	30	0.755 ± 0.052	**3.45^{b}± 0.28**
NO_3 starvation	NH_4Cl	180	**0.484^{b}± 0.140**	**2.92^{a}± 0.84**
P-starvation				
Full nutrition	none	0	0.198 ± 0.120^{d}	n.d.
PO_4 starvation	none	0	0.257 ± 0.060	n.d.
PO_4 starvation	KH_2PO_4	180	0.330 ± 0.098	n.d.
S-starvation				
Full nutrition	none	0	0.686 ± 0.221	**5.92^{a}± 0.59**

SO_4 starvation	none	0	0.618 ± 0.100	5.03 ± 0.63
SO_4 starvation	KCl	30	0.750 ± 0.202	6.05 ± 1.28
SO_4 starvation	KCl	180	0.616 ± 0.136	4.11 ± 1.18
SO_4 starvation	K_2SO_4	30	0.545 ± 0.118	5.09 ± 0.95
SO_4 starvation	K_2SO_4	180	0.522 ± 0.085	4.81 ± 0.69

Supporting Table S2. Effect of α-amanitin and cordycepin on transcript levels of sucrose-inducible genes. C-starved 9-d-old *Arabidopsis thaliana* seedlings were incubated with 20 µM α-amanitin or 0.6 mM cordycepin for 1h before addition of sucrose (15 mM final concentration). Transcript abundance was measured by RT-qPCR. Data are expressed as 40-ΔC_t with *GAPDH* (At1g13440) as the reference gene. Values are mean ± SD (*n*=4).

**Time after sucrose addition (*min*)**	**40-ΔC_t**		
	**No inhibitor**	**20 µM α-amanitin**	**0.6 mM cordycepin**
At1g17745 (3PG-DH)			
0	35.1 ± 0.3	35.5 ± 0.2	35.3 ± 0.2
30	35.4 ± 0.3	35.7 ± 0.3	35.0 ± 0.3
180	36.6 ± 0.1	37.0 ± 0.1	35.4 ± 0.2
At1g61800 (GPT2)			
0	34.0 ± 0.3	32.9 ± 1.2	30.9 ± 0.4
30	35.4 ± 0.3	33.7 ± 1.2	30.5 ± 0.4
180	39.2 ± 0.2	36.0 ± 0.4	30.4 ± 0.9
At3g12580 (HSP70)			
0	34.5 ± 0.7	34.2 ± 0.6	32.8 ± 0.4
30	34.8 ± 0.7	35.5 ± 1.1	32.7 ± 0.4
180	37.5 ± 0.4	37.8 ± 0.2	34.5 ± 0.9
At5g24660 (unknown)			
0	30.8 ± 0.2	30.2 ± 0.4	27.7 ± 0.2
30	33.1 ± 0.5	33.1 ± 0.5	29. 1± 0.2
180	34.1 ± 0.0	34.1 ± 0.0	29.7 ± 0.2
At5g48570 (PPCTI)			
0	32.2 ± 0.6	32.4 ± 0.6	32.0 ± 0.7
30	32.6 ± 0.7	32.8 ± 0.6	31.7 ± 0.3
180	34.2 ± 0.7	34.4 ± 0.3	32.9 ± 0.4

Supporting Table S3. Effect of sucrose resupply on ribosomal occupancy of *TPS*, *TPP* and *TRE* transcripts in C-starved *Arabidopsis thaliana* seedlings. C-starved 9-d-old seedlings were supplied with 15 mM sucrose. Free mRNAs and monosomes (non-polysomal fraction; NPS) were separated from polysomes (polysomal fraction; PS) by sucrose density gradient centrifugation and transcripts were quantified in each fraction by Real-Time RT-qPCR. Ribosomal occupancy was calculated as the ratio of transcript levels in PS/(NPS+PS). Data are mean ± S.D. (n = 3[a] or 2[b]). n.d., transcript not detected in one or both fractions. [c]Significantly different from C-starved control according to Student's t-test (p<0.05).

**Gene**	**Locus**	**(%) ribosomal occupancy**		
		**C-starved[a]**	**Suc 30 min[b]**	**Suc 3 h[a]**
TPS1	At1g78580	85 ± 7	87 ± 0	87 ± 5
TPS2	At1g16980	n.d.	n.d.	n.d.
TPS3	At1g17000	n.d.	n.d.	n.d.
TPS4	At4g27550	n.d.	n.d.	75 ±10
TPS5	At4g17770	92 ± 4	85 ± 8	91 ± 5
TPS6	At1g68020	88 ± 6	95 ± 0	89 ± 5
TPS7	At1g06410	92 ± 6	96 ± 0	94 ± 3
TPS8	At1g70290	78 ± 7	88 ± 7	76 ±10
TPS9	At1g23870	82 ± 8	88 ± 0	81 ±12
TPS10	At1g60140	73 ± 3	77 ±10	66 ±17
TPS11	At2g18700	85 ± 6	92 ± 5	87 ± 4
TPPA	At5g51460	74 ± 5	77 ± 5	73 ± 5
TPPB	At1g78090	86 ±13	79 ± 7	78 ± 1
TPPC	At1g22210	n.d.	73 ±16	75 ± 8
TPPD	At1g35910	84 ±16	71 ± 9	84 ± 6
TPPE	At2g22190	74 ±10	79 ± 2	80 ± 5
TPPF	At4g12430	71 ±11	76 ± 1	79 ± 5
TPPG	At4g22590	75 ± 3	79 ± 5	79 ± 2
TPPH	At4g39770	78 ± 3	76 ± 7	79 ± 2

TPPI	At5g10100	73±5	68 ± 7	67 ± 6
TPPJ	At5g65140	89 ± 9	70 ± 5	72 ± 4[c]
Trehalase	At4g24040	88 ± 3	90 ± 3	85 ± 2
UBQ10	At4g05320	78 ± 5	78 ± 5	80 ± 2
GAPDH	At1g13440	73 ±13	91 ± 2	92 ± 2

Supporting Table S4. Analysis of commercially supplied trehalose-6-phosphate. Trehalose-6-phosphate (dipotassium salt; "95% purity"; Lot 124K3787) was obtained from Sigma-Aldrich Chemie GmbH (Taufkirchen, Germany). A nominally 1 mM solution in water was analysed by Fourier transform mass spectrometry in negative ion mode (Giavalisco et al., 2008, *Anal. Chem* . 80; 9417-25). Compound assignments are based on the measured m/z, with superscripts showing alternative assignments based on monovalent (-1) and divalent (-2) ions. Potential isomers are given in the right hand column. The signal intensity was used to estimate the percentage contribution of each compound to the weight of the original material.

Compound	Formula	Mass	Charge	*m/z*	Intensity	Weight %
trehalose 6-phosphate	$C_{12}H_{23}O_{14}P$	422.28	-2	210.03	4580691	60.7
palmitic acid	$C_{16}H_{32}O_2$	256.42	-1	255.23	928980	7.5
stearic acid	$C_{18}H_{36}O_2$	284.48	-1	283.26	825808	7.4
hexose-phosphate	$C_6H_{13}O_9P$	260.14	-1	259.02	843734	6.9
oleic acid	$C_{18}H_{34}O_2$	282.46	-1	281.25	437636	3.9
myo -inositol 1,2-cyclic phosphate	$C_6H_{11}O_8P$	242.02	-1	241.01	323344	2.5
hexadecenoic acid	$C_{16}H_{30}O_2$	254.41	-1	253.22	219412	1.8
α,α-trehalose	$C_{12}H_{22}O_{11}$	342.3	-1	341.11	110766	1.2
pentose-phosphate	$C_5H_{11}O_8P$	230.11	-1	229.01	128991	0.9
linoleic acid	$C_{18}H_{32}O_2$	280.45	-1	279.23	102567	0.9
lignoceric acid	$C_{24}H_{48}O_2$	368.64	-1	367.36	74045	0.9
myristic acid	$C_{14}H_{28}O_2$	228.37	-1	227.20	110571	0.8
isopropyl myristate	$C_{17}H_{34}O_2$	270.45	-1	269.25	66333	0.6
docosanoic acid	$C_{22}H_{44}O_2$	340.58	-1	339.33	32403	0.3
cyclohexaneundecanoic acid	$C_{17}H_{32}O_2$	268.43	-1	267.23	39308	0.3
arachidic acid	$C_{20}H_{40}O_2$	312.53	-1	311.30	28905	0.3
laurocapram	$C_{18}H_{35}NO$	281.48	-1	280.26	26197	0.2
pelargonic acid[a]	$C_9H_{18}O_2$	158.24	-1	157.12	43952	0.2
dihydroxystearic acid[a]	$C_{18}H_{36}O_4$	316.48	-2	157.12	43952	(0.4)
capric acid[b]	$C_{10}H_{20}O_2$	172.26	-1	171.14	39334	0.2
Solutol HS 15[b]	$C_{20}H_{40}O_4$	344.53	-2	171.14	39334	(0.4)

ethyl oleate	$C_{20}H_{38}O_2$	310.51	-1	309.28	20038	0.2
oxooctadecanoic acid	$C_{18}H_{34}O_3$	298.46	-1	297.24	19208	0.2
deacetylcephalosporin C	$C_{14}H_{19}N_3O_7S$	373.38	-1	372.09	14158	0.2
oxononanoic acid	$C_9H_{16}O_3$	172.22	-1	171.10	28685	0.2
ethyl linoleate	$C_{20}H_{36}O_2$	308.5	-1	307.26	15477	0.1
isopropyl palmitate	$C_{19}H_{38}O_2$	298.5	-1	297.28	15331	0.1
bis(2-ethylhexyl) adipate	$C_{22}H_{42}O_4$	370.57	-1	369.30	10287	0.1
2-hydroxystearate	$C_{18}H_{36}O_3$	300.48	-1	299.26	12285	0.1
farnesal	$C_{15}H_{24}O$	220.35	-1	219.18	16140	0.1
DHAP	$C_7H_{13}O_{10}P$	288.15	-1	287.02	12080	0.1
methyl oleate	$C_{19}H_{36}O_2$	296.49	-1	295.26	11099	0.1
Sulbactam(antibiotic)	$C_8H_{11}NO_5S$	233.24	-1	232.03	12817	0.1
L-phenylalanine	$C_9H_{11}NO_2$	165.19	-1	164.07	17949	0.1
myristoleic acid	$C_{14}H_{26}O_2$	226.36	-1	225.19	12270	0.1
mevaldic acid	$C_6H_{10}O_4$	146.14	-1	145.05	18365	0.1
hexose sugar or *myo* -inositol[c]	$C_6H_{12}O_6$	180.16	-2	89.02	14878	0.1
lactic acid[c]	$C_3H_6O_3$	90.08	-1	89.02	14878	(0.1)
caprylic acid	$C_8H_{16}O_2$	144.21	-1	143.11	18380	0.1
suberic acid	$C_8H_{14}O_4$	174.19	-1	173.08	14185	0.1
sulphuric acid (as HSO_4^- ion)	H_2SO_4	98.08	-1	96.96	24802	0.1
4-hydroxybutyric acid	$C_4H_8O_3$	104.1	-1	103.04	20261	0.1
ethyl oxohexanoate	$C_8H_{14}O_3$	158.19	-1	157.09	11550	0.1
meglutol[d]	$C_6H_{10}O_5$	162.14	-1	161.05	10998	0.1
bis-D-fructose 2',1:2,1'-dianhydride[d]	$C_{12}H_{20}O_{10}$	324.28	-2	161.05	10998	(0.1)
L- histidine	$C_6H_9N_3O_2$	155.15	-1	154.06	11553	0.1
acetyl phosphate	$C_2H_5O_5P$	140.03	-1	138.98	10623	<0.1
phosphoric acid (as $H_2PO_4^-$ ion)	H_3PO_4	98	-1	96.97	10007	<0.1

m/*z*	**Alternative identifications and notes**
210.03	sucrose-6-phosphate; sucrose-6´-phosphate; maltose-1-phosphate
255.23	
283.26	
259.02	e.g. D-fructose 6-phosphate; D-glucose 6-phosphate; D-glucose alpha-1-phosphate; galactose 6-phosphate
281.25	
241.01	
253.22	
341.11	sucrose, maltose or other disaccahride sugar
229.01	e.g. ribose 5-phosphate; D-ribulose 5-phosphate; ribose 1-phosphate; L-ribulose 5-phosphate;
279.23	
367.36	
227.20	
269.25	
339.33	
267.23	
311.30	
280.26	
157.12	
157.12	
171.14	isoamyl isovalerate; ethyl caprylate;
171.14	*synthetic non-ionic solubiliser and emulsifying agent*

309.28	
297.24	hydroxyoctadecenoic acid; epoxyoctadecanoic acid; rosaprostol; methyl palmoxirate
372.09	*antibiotic*
171.10	
307.26	sclareol
297.28	
369.30	*plasticizer*
299.26	
219.18	α-santalol
287.02	*3-deoxy-D-arabino-heptulosonic acid 7-phosphate*
295.26	lactobacillic acid
232.03	dopamine 3-O-sulfate; dopamine 4-O-sulfate;
164.07	D-phenylalanine; Benzocaine
225.19	
145.05	
89.02	e.g. glucose, fructose, galactose
89.02	
143.11	valproic acid
173.08	dimethyl adipate
96.96	
103.04	
157.09	
161.05	3,3-dimethylmalate
161.05	
154.06	
138.98	
96.97	

Supporting Table S5. Primers used for Real-Time RT-qPCR analysis. Primers were designed using the Primer Express 2.0 software package (www.appliedbiosystems.com) acccording to the following criteria: annealing temperature 60±2°C, primer length of 18-24 nucleotides, GC content 35-45%, and amplicon lengths of 60-150 bp. Primer sequences were compared with the *Arabidopsis thaliana* genome sequence using the BLAST algorithm (http://www.arabidopsis.org/Blast) to check their specificity. In addition, the specificity of the primers was verified by checking the dissociation curve from the RT-qPCR analysis and that the reaction gave a single amplicon of the expected size. The PCR efficiencies of primers were estimated using the LinRegPCR software (Ramakers *et al.*, 2003). *TPP* gene nomenclature is in accordance with Vandesteene *et al*. (2012). All primer sequences are shown 5´→3´.

Gene	Locus	Forward primer	Reverse primer
TPS1	At1g78580	GGCCCTTAAAGGCATTATGCA	GCTGCTTCCGCTCAGAACAA
TPS2	At1g16980	GAAAATTCGAAACGCTGGATCA	GCCGATTTGCCCAGTGTCT
TPS3	At1g17000	TGTCGCCCAAGTTATCTCATGA	GCGAGCTTGTCCTATGGCTG
TPS4	At4g27550	CCAGAGTTGACCAAGAAAGCCA	TGGTCGATGATACCTTCTTGGG
TPS5	At4g17770	CTGCTCTGATGCTCCTTCTTCC	AAGCTGGTTTCCAACGATGATG
TPS6	At1g68020	AGCATGGACGAAGGAGGTGTT	CCTGAAGCTCTGGTCTAGTGCC
TPS7	At1g06410	GGCAGTGAAGAATGGGAGACAT	TGCTTCATTACAGGTTCCACGA
TPS8	At1g70290	TGGAGACTCATCATCGAGCTTAAA	GCTTTCAAATGCAACTTGTGTGTG
TPS9	At1g23870	CTGCAACGAGCAGCTCGAAG	CTCAAACGCCACTTGCGTGT

TPS10	At1g60140	TGTGGGAAGAAAACCGAGCA	CCTTGAAGCAACTTCACCACGT
TPS11	At2g18700	CGCCACCAGAAGCATCATAAGT	TCTTGGTCATAACTCCGCGC
TPPA	At5g51460	TGTGATTGACTGGGACAAAGGG	CCTCACAGTTGTTTAGGCCGAG
TPPB	At1g78090	GGAGAAGAGTGAAAGACATGCAAC	CACCCTCCAGGCATCTACTAATTT
TPPC	At1g22210	GCGTTTGGTAAAGTGGAAGAAACA	TCCCATATATTTCAGGACGGGTTA
TPPD	At1g35910	TCCAAAATTCCAAAGGAAACGA	TGCAAAAACTCTCCTACCTCGG
TPPE	At2g22190	CAAAGGCCATCTCAAACTCGG	CGTTGGTGACGTGTCTCCGT
TPPF	At4g12430	TTAACCAATCAAGACTCGGCGT	TCATCGAGTTTCGCAGGTTTCT
TPPG	At4g22590	GAAGCTTCATTCTGCACTGCTTTT	CCCGTTTGCTTAATGGATAATATGG
TPPH	At4g39770	GACTAGCGCGTCTTATTCACTGC	TCCATTCCACTAAACGTTGCAAG
TPPI	At5g10100	CAAATGCAGCCAAGAATGTGAA	GGCATTTCGAATATTGTCACTCGT
TPPJ	At5g65140	GACGGTTGGTGGAGTGGAAA	AAATGTACTTCATTGCCCCTTGC
Trehalase	At4g24040	GACAACAATGGGATTCTCCGAA	TCGATCTTCCGAGCCCTGT
**Reference genes**			
GAPDH(5´)	At1g13440	TCTCGATCTCAATTTCGCAAAA	CGAAACCGTTGATTCCGATTC
GAPDH(3´)	At1g13440	TTGGTGACAACAGGTCAAGCA	AAACTTGTCGCTCAATGCAATC
GAPDH	At1g13440	TTGGTGACAACAGGTCAAGCA	AAACTTGTCGCTCAATGCAATC
UBQ10	At4g05320	GGCCTTGTATAATCCCTGATGAATAAG	AAAGAGATAACAGGAACGGAAACATAGT
MADS	At5g65050	TAGCCAGGTGGGGAAGAAGA	TTTGTTGCCGGAGCCATTTT

ACTIN2	At3g18780	ACTTTCATCAGCCGTTTTGA	ACGATTGGTTGAATATCATCAG
**Sucrose induced genes**			
3PG-DH	At1g17745	TGGTTGTTGATTCATCACCAGAGTACCCTG	TAAATGCGGAACTCCGTACTTCACCTTCCC
GPT2	At1g61800	AGCTTAACTTCAACATCACTGGGTTTATGG	TCATCGATAAGCAAGCGTAGTAGTTCATTC
HSP70	At3g12580	GGTCAGAAGAACAAGATCACAATCACAAAC	CCATCTTCTCGATCTCTTCCTTTGATAACC
unknown	*At5g24660*	AAGTGGACGAGCTACGACGGAAGAAC	GCAACATCTCTTTCTTCATCTCCTCCACAG
PPCTI	At5g48570	CAAGCTGAAACTGAAAGATTACAAGGAAGC	CCTATACATTGCCTTCACGTTCCTACTATC
